# An in vivo brain–bacteria interface: the developing brain as a key regulator of innate immunity

**DOI:** 10.1038/s41536-020-0087-2

**Published:** 2020-02-04

**Authors:** Celia Herrera-Rincon, Jean-Francois Paré, Christopher J. Martyniuk, Sophia K. Jannetty, Christina Harrison, Alina Fischer, Alexandre Dinis, Vishal Keshari, Richard Novak, Michael Levin

**Affiliations:** 1 Allen Discovery Center, and Department of Biology, Tufts University, Medford, MA USA; 2 Center for Environmental and Human Toxicology and Department of Physiological Sciences, University of Florida, Gainesville, FL USA; 3 Wyss Institute for Biologically Inspired Engineering, Harvard University, Boston, MA USA

**Keywords:** Neuroscience, Immunology

## Abstract

Infections have numerous effects on the brain. However, possible roles of the brain in protecting against infection, and the developmental origin and role of brain signaling in immune response, are largely unknown. We exploited a unique *Xenopus* embryonic model to reveal control of innate immune response to pathogenic *E. coli* by the developing brain. Using survival assays, morphological analysis of innate immune cells and apoptosis, and RNA-seq, we analyzed combinations of infection, brain removal, and tail-regenerative response. Without a brain, survival of embryos injected with bacteria decreased significantly. The protective effect of the developing brain was mediated by decrease of the infection-induced damage and of apoptosis, and increase of macrophage migration, as well as suppression of the transcriptional consequences of the infection, all of which decrease susceptibility to pathogen. Functional and pharmacological assays implicated dopamine signaling in the bacteria–brain–immune crosstalk. Our data establish a model that reveals the very early brain to be a central player in innate immunity, identify the developmental origins of brain–immune interactions, and suggest several targets for immune therapies.

## Introduction

Innate immunity provides a first line of defense against pathogens^1^ and plays a crucial role in initiating adaptive immune responses.^2^ Most of the cell types of hematopoietic origin and genes known to be involved in mammalian innate and adaptive immunity have been identified in *X. laevis*,^3,4^ making the frog model an important emerging tool for understanding the cell and molecular biology of immunity.^1,5,6^ The immune response in the *Xenopus* embryo is provided uniquely by innate immunity during the first 12 days (d) post-fertilization,^7^ allowing studies of response to infection by wide range of pathogens without the confounding influences of adaptive immune component.^5,8–11^

An important emerging field concerns bidirectional intercommunication between two ‘super-systems’, the immune response and the brain, with implications for both basic evolutionary/cell biology and for biomedicine.^12–14^ Cytokines and other inflammation-related molecules affect vagal afferents or directly on brain, controlling aspects of behavior.^15^ Exciting recent studies integrating cognitive endpoints and cell biology of lymphatic vessels in the mature brain reveal the link between immune cells and brain functions.^2,16–19^ These studies reveal the adult brain as a regulator of adaptive immune response.^20,21^ The immune system has been proposed as a ‘seventh sense’,^22^ receiving information from pathogen agents to inform the central nervous system. Studies using invertebrate models suggest that neural circuits receive and integrate stimuli coming from pathogens, via G protein-coupled receptors (GPCRs), to guide the immune response.^23–25^

Several key open questions remain in this fascinating field. First, while much work has been done on the role of the CNS in adaptive immunity, the interplay between brain and *innate* immunity is still poorly understood. Second, most of the data come from adult organisms, and the developmental origins of brain–immune interactions are largely mysterious. Last, an obvious limitation of mammalian models is the difficulty of performing loss-of-function studies that unequivocally show that the brain controls the immune system.^26^ To address these knowledge gaps, and to identify intervention strategies for innate immune function, we exploited a unique system for probing the interactions of brain and immunity in embryogenesis under normal conditions and when challenged with human pathogenic bacteria.

We developed a *Xenopus* model in which we could study brain-dependent events in embryogenesis: the brain is removed during early embryonic stages, but the animal can be kept alive and development continues. The ability of this vertebrate, a popular model for numerous biomedical contexts,^27–34^ to survive and develop without a brain provides a unique opportunity to understand the role of the brain in diverse systems-level outcomes. Our prior research into brain-dependent developmental signaling revealed that the nascent brain, even before being fully formed, plays an instructive role in patterning somitic muscle and peripheral neural networks.^35,36^ Here, we use this brainless vertebrate model, with intact spinal cord and peripheral innervation, to demonstrate an unknown role of the brain: regulating the early innate immunity in the presence or absence of infection. Our data, synthesizing a molecular comparison of infected and uninfected animals under normal, brainless, and tail-regenerative conditions, reveal the profound influence of the brain, in part mediated by dopamine signaling, upon susceptibility and response to pathogenic challenge at the cellular, molecular, and organism-wide levels.

## Results

### Having a brain protects against infections

**Fig. 1:**
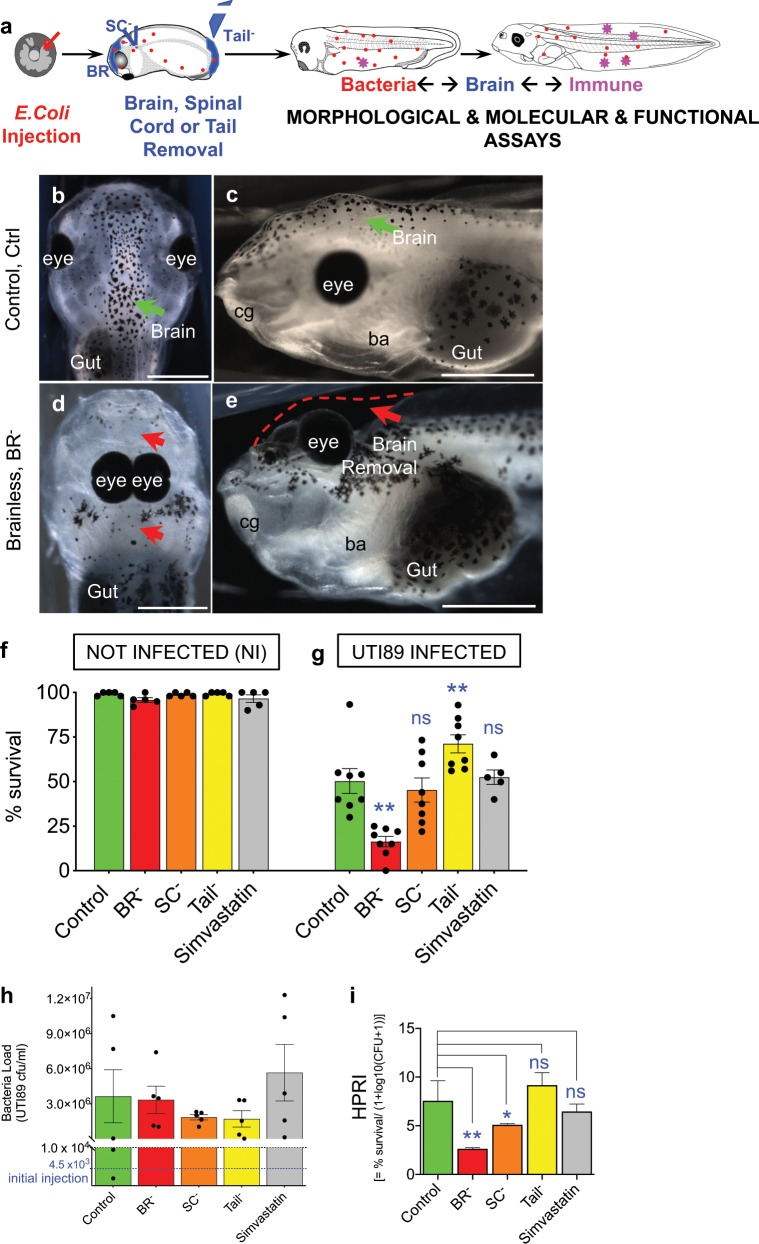
The presence of a brain protects against systemic infection in *Xenopus* embryos. **a** One day after fertilization (early- to mid-gastrula or stage 12), *Xenopus* embryos were microinjected with the pathogenic bacteria *E. coli* UTI89. The next day, surgeries were performed for removal of the brain (brainless or BR^–^ embryos), a piece of cervical spinal cord (SC^–^ embryos) or the tail bud (Tail^–^ embryos). Embryos were collected for morphological and molecular analysis during the next three days post-surgery. The bacterial load is represented in red. **b**–**e** Dorsal (left column) and lateral (right column) views of st. 48 embryos belonging Control or Intact (Ctrl; **b**, **c**) vs. Brainless (BR^−^; **d**, **e**) experimental groups. Green and red arrows point, respectively, the control or correct vs. aberrant morphologies after surgery removal. Eyes, gut and branchial arches (ba) are indicated for reference. Left: rostral is up. Right: rostral is left, dorsal is up. Rostral is left and dorsal is up. Scale bar = 500 μm. **f**, **g** Survival rates (plotted as percentage, %) of each experimental group per each infection condition: without *E. coli* infection or not-infected animals (NI; **f**, one-way ANOVA *P* > 0.05) vs. *E. coli* UTI89 infected animals (UTI, evaluated four days after infection; **g**, one-way ANOVA *P* < 0.01). Data represent the mean and S.D. of, at least, five independent replicates. Each replicate is shown by one dot. **h** Bacteria load measured and plotted as colony forming units per milliliter (cfu/ml) in independent embryos (dots) belonging each experimental group or surgery condition. Alive embryos were harvested for analysis 48 h after infection. Initial bacteria load or number of bacteria injected (average of three independent replicates) at *t* = 0 is plotted as a blue-dashed line. One-way ANOVA *P* > 0.05. **i** Host-Pathogen Response Index (HPRI) = % survival/(1 + log10(CFU + 1)), for each experimental group, as a metric of tolerance. Data represent the mean and S.D. of five independent embryos. One-way ANOVA *P* < 0.01. **g**, **i** *P* values after post hoc Bonferroni comparisons are indicated as **P *<* 0.01, *P *<* 0.05, ns *P* > 0.05. See also Supplementary Fig. S1.

We previously showed that the uropathogenic *E. coli* UTI89 readily colonizes *X. laevis* embryos when infected at blastula or gastrula stages,^9^ and that survival rates at 4–5 days after infection could be used as a readout of the degree of activation of the innate immune system. Here, we investigated the role of brain-derived signals in innate immunity and susceptibility by asking how animals respond to systemic infection in the absence of a brain (Fig. 1a).

Bacteria were injected into the blastocoel of embryos at 4.5 × 10^3^ ± 7.8 × 10^3^ cfu/embryo. First, we evaluated the survival rates at stage 48 (4–5 d after infection) for embryos subjected to the different infection conditions: infected with *E. coli* UTI89 (UTI condition) vs. not-infected (NI condition), belonging to each intervention or experimental group at st. 25 (Fig. 1b–e, Supplementary Fig. S1): intact brain or control embryos (Control or Ctrl; Fig. 1b, c), brain removal (brainless or BR^−^; Fig. 1d, e; Supplementary Fig. S1a, b), resection of a piece of cervical spinal cord (SC^–^; Supplementary Fig. S1c, d), tail-bud amputation (Tailless or Tail^–^), and muscle lysis via Simvastatin treatment (Simv; Supplementary Fig. S1h, j). At least, five biological replicates or dish of embryos (*r* = 5, *n* = 40 embryos per replicate, *N* total = 200 embryos) were used per each group. Results were consistent among the replicates for each condition and group. For NI embryos, survival rates were similar among groups, ranging from 92 to 100% (99 ± 1% for Ctrl, 96 ± 3% for BR^−^, 99 ± 1% for SC^−^, 99 ± 1% for Tail^−^, and 97 ± 5% for Simv; one-way ANOVA *P* > 0.05; *r* = 5 per group; Fig. 1f), suggesting any further differences among groups cannot be due to the surgical intervention itself. *E. coli* infection caused significant differences in the survival rates among groups (one-way ANOVA *P* < 0.01; Fig. 1g), indicating its utility as infection-survival assay. For intact or Ctrl embryos the mean infection-survival rate was 50 ± 20% (*r* = 8). Tail^–^ animals expressed significantly higher survival rates with respect to Ctrl (71 ± 14%, Bonferroni’s posttest *P* = 0.018, *r* = 8), apparently resulting from the induced regenerative response.^9,37^ Conversely, infected animals developing without a brain (BR^–^) exhibited a low survival rate, with a significant drop to 16 ± 8% (Bonferroni’s posttest *P* < 0.0001, *r* = 8). The survival rate for SC^–^ embryos (37 ± 15%) was not significantly different than Ctrl group (*P* > 0.9999, *r* = 8), indicating that the effect was not due simply to surgical damage or the requirement for intact CNS overall. As a control for possible effects of general tissue distress on outcomes, and to ascertain potential impact of muscle structure on survival phenotypes,^36^ we tested Simvastatin treatment (Simv; Supplementary Fig. S1h–j). Simvastatin is a drug well-known to give severe collateral myotoxic effects in human patients^38^ and zebrafish embryos.^39,40^ It induces muscle disorganization similar to that observed after brain removal in *Xenopus* embryos, allowing independent characterization of the effects of brain removal vs. of muscle tissue lysis and disorganization. Simv group’s survival was similar to that of Ctrl (52 ± 9%; *P* > 0.9999, *r* = 5), demonstrating that the survival capacity and susceptibility to bacterial infection is not non-specifically modulated by a stressful combination of infection and strong tissue stressor.

We next wanted to ensure that the effects of the brain ablation were not due to the removal of migrating neural crest cells (NC; Supplementary Fig. S1e–g). Survival after NC ablation in absence of infection was similar to the other groups, 98 ± 3% (*r* = 3, *n* = 40, *N* = 120), without any significant death related to the intervention itself. UTI infection in NC^–^ embryos lead to a mean survival rate of 60 ± 13% (*r* = 3, *n* = 40, *N* = 120), similar to that reached in Ctrl animals (61 ± 30%; *r* = 3, *n* = 40, *N* = 120) and significantly different from BR^–^ group (11 ± 12%; *r* = 3, *n* = 40, *N* = 120; one-way ANOVA *P* < 0.05; Bonferroni’s test for NC^–^ vs. Ctrl *P* *=* 0.9365; NC^−^ vs. BR^–^ *P* = 0.458). Of the several tissues and organs we targeted, surgically and via lytic toxins, the brain was unique in its effects in promoting survival.

To further characterize the role of brain in modulating overall susceptibility to bacterial infection, we assessed the bacterial load in living embryos within each experimental group at 2 d after infection. Microbial analysis showed that the pathogen load was maintained among the different experimental groups, with an average of 3.24 × 10^6^ cfu/ml (one-way ANOVA *P* > 0.05; Fig. 1h: the average initial dose of UTI *E. coli* injected was 4.5 × 10^3^ cfu/ml). Since the presence of brain improves survival but does not significantly reduce pathogen load, we analyzed our groups with an HPRI metric—an index that takes into account pathogen load as well as survival (Fig. 1i). Differences in HPRI index demonstrated that developing without an intact Central Nervous System (CNS; brain or spinal cord) significantly decreases tolerance^41,42^ and increases the susceptibility to infection (one-way ANOVA *P* < 0.01).

Taken together, the results demonstrate the unique effects of brain removal on susceptibility to infection: survival depends on the presence of the brain regardless of any tissue injury, and the absence of brain makes embryos likely to succumb to bacterial infection.

### Early brain protects against the infection-induced apoptosis

**Fig. 2:**
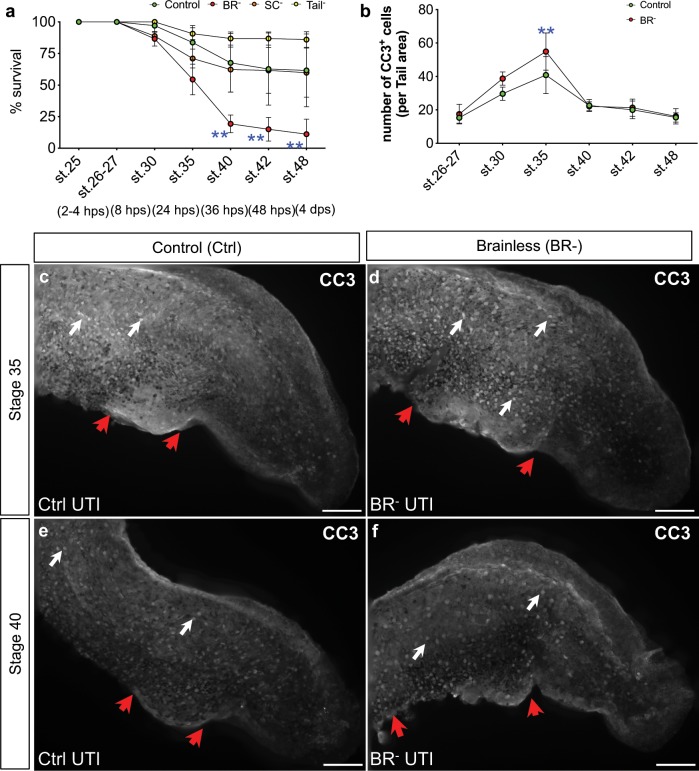
The presence of a brain protects against infection-induced death, which is more severe during the first 48 h post infection. **a** Longitudinal assay, scoring survival rates after infection for Control (Ctrl, green), Brainless (BR^–^, red), Spinal Cord Resection (SC^–^, orange) and Tailless (Tail^–^, yellow) embryos at 2–4 h post-surgery (hps; corresponding to st. 26–27), 8 hps (st. 30), 24 hps (st. 35), 36 hps (st. 40), 48 hps (st. 42) and the end stage-48 time point (4 days ps). Data represent the mean and S.D. of three independent replicates. **b**–**f** Number (**b**) and expression pattern (**c**–**f**) of apoptotic cells (positively reacted against Cleaved Caspase-3 Asp175 antibody; CC3) in Ctrl (green) and BR^–^ (red) animals at the same longitudinal period than (**a**). Data represent the mean and S.D. of, at least, ten embryos coming from three different replicates. **c**–**f** Lateral views after CC3 immunofluorescence of UTI-infected Ctrl (**c**, **e**) and BR^–^ (**d**, **f**) embryos at st. 35 (**c**, **d**) and st. 40 (**e**, **f**). White arrows point CC3-expressing cells; red arrows indicate damage related to the infection along the gut area. Rostral is left and dorsal is up. Scale bar = 250 μm. **a**, **b** Two-way ANOVA *P* < 0.01. *P* values after post hoc Bonferroni comparisons are indicated as **P *<* 0.01.

To understand the increased susceptibility to infection in BR^–^ animals, we conducted a longitudinal assay, scoring survival rates and characterizing apoptosis at several time points after infection and surgery (*r* = 3, *N* = 120 per group; Fig. 2a, b). Embryos were analyzed at 2–4 h post-surgery (hps; corresponding to st. 26–27), 8 hps (st. 30), 24 hps (st. 35), 36 hps (st. 40), 48 hps (st. 42) and the end stage-48 time point (4 days ps). For all groups, the first wave of death was detected at 24 hps (or 36 h post infection, hpi; Supplementary Table S1), specially for BR^–^ animals, which went from 87 ± 6% at st. 30 to 54 ± 12% at st. 35 (two-way ANOVA *P* < 0.01; Bonferroni’s posttest *P* = 0.0005 for BR^–^ st. 25 vs. st. 35). For Ctrl animals, this peak (or first time point with significant differences respect to st. 25) was reached at st. 40 (48 hpi), with a drop in the survival rate to 68 ± 23%. At st. 40, BR^–^ animals showed a dramatic decreased survival rate (19 ± 7%), leading to significant differences with the rest of the groups, which kept constant until the end of the experiment (Bonferroni’s posttest *P* < 0.01 for BR^–^ vs. C; see Supplementary Table S1 for over-time values at each stage and per each group). To characterize the cellular mechanisms behind the infection-induced death, we studied the patterning and number of apoptotic cells in Ctrl and BR^–^ animals at the same longitudinal period (Fig. 2b). We used the antibody against Cleaved Caspase-3 (Asp175; CC3, which detects levels of activated caspase-3) as it has been extensively shown to be a reliable marker for apoptosis in *Xenopus* embryos.^43–45^ For both Ctrl and BR^–^ groups, apoptosis followed a successive positive slope from st. 26–27, reaching a peak at st. 35, when significant differences are found in the number of cells between them (41 ± 11 vs. 55 ± 11 CC3-positive cells per area in Ctrl vs. BR^–^, respectively; two-way ANOVA *P* < 0.01; Bonferroni’s posttest *P* = 0.0018; Fig. 2c, d). Morphological analysis of CC3 expression revealed that UTI Infection seems to affect mostly the gut region, characterized by a focus of highly reactivity to CC3 marker, which is focused on a posterior domain in Ctrl animals and occupying a more extensive area in BR^–^ embryos (Fig. 2c, d). Animals at later stages in both groups expressed abnormalities in this region, but with decreased apoptosis, pointing this damage on the digestive domain as indicative of the UTI infection and defining the lethal consequences of infection to occur during the first 48 hpi. From st. 35, apoptosis starts to decrease, being constant and without differences between groups until the end of the experiment (Fig. 2e, f). Taking together, these results show that death after infection-surgery occurs sequentially over the time, as a direct consequence of increased weakness, and it does not happen at the first immediate hours after surgery (st. 26–30). Interestingly, this analysis also shows that the peak of death in absence of brain occurs 24 h earlier than when the brain is present (at st. 35 vs. st. 40), with a significant increased apoptosis, supporting the protective effects of brain against the lethal consequences of the infection.

### Brain signaling does not affect primitive myeloid precursors

Next, we asked whether the protective effects of the brain were due to action on the development of the primitive immune system (regardless of infection, as it does with muscle and nerve development) or whether, conversely, the brain acted on the active response to infection (implying differences between Control and BR^–^ animals only after infection). In *Xenopus*, the earliest known marker of the primitive myeloid is *spib-a* (*spiba*). Spib- is a highly conserved ETS transcription factor that marks the primitive myeloid-cell lineage and is required for its development.^3,10^ *spib* expression in myeloid precursors has been characterized in *Xenopus*,^10^ mouse,^46^ and humans.^47^ To specifically probe the role of the brain in establishing key cell types involved in innate immunity, we studied the *spiba*-positive^10^ cells in UTI and NI early-staged embryos (st. 28), subjected to the different surgical interventions (Ctrl, BR^–^, SC^–^, Tail^–^; Supplementary Fig. S2).

To compare the prevalence and spatial distribution of the *spiba*-positive cell population, we characterized four independent regions: face, tail, dorsal flank, and ventral flank. At least, ten different embryos, from different replicates, per each group and condition were used for quantification (*r* = 3, *n* = 40, *N* = 120; Supplementary Fig. S2a, b, t–w and Methods for details of quantification). NI embryos belonging to each experimental group possessed a similar number and distribution of positive cells, mainly located in the ventral region or hematopoietic organ at these stages (Supplementary Fig. S2c, d). In NI embryos, the only intervention that resulted in significant differences at the site injury from intact embryos was tail-bud amputation (one-way ANOVA *P* < 0.05; Bonferroni’s posttest *P* = 0.032; Supplementary Fig. S2f). The number of *spiba*^*+*^ cells in Tail^–^ animals was especially higher in the posterior regions, occupying the bud and amputation plane. No differences from Ctrl embryos were detected for any area in BR^–^ animals, suggesting the brain, in the absence of infection, is not necessary to maintain this population of primitive myeloid precursors.

Next, we analyzed the same cell population in embryos that were previously infected with *E. coli* UTI89 (UTI condition; Supplementary Fig. S2j–n). Neither intervention (brain, SC or tail removal) nor body region showed significant differences in number of *spiba*^+^ cells within infected embryos. Comparing effects of the infection on number of cells within each experimental group (Supplementary Fig. S2o–r), we detected a significant decrease in the total number of myeloid precursors as consequence of bacteria injection for all experimental conditions (two-way ANOVA *P* < 0.05). This generic drop detected in st. 28 embryos indicates that infection with the human uropathogenic *E. coli* affects the primitive myeloid cells at early stages of immune system development. Intriguingly, tail amputation performed after infection, which showed the highest survival rates, did not induce a higher proliferation of *spiba*^*+*^ cells, suggesting that the higher survival percentage observed in this group might not due to the presence of more myeloid progenitor cells.

Taken together, our results reveal the negative impact of infection at very early embryonic stages and demonstrate that the positive contribution of the brain to surviving infection is not mediated by brain-induced changes in the *number* of early myeloid-cell precursors.

### Brain is required for migratory response of macrophages

**Fig. 3:**
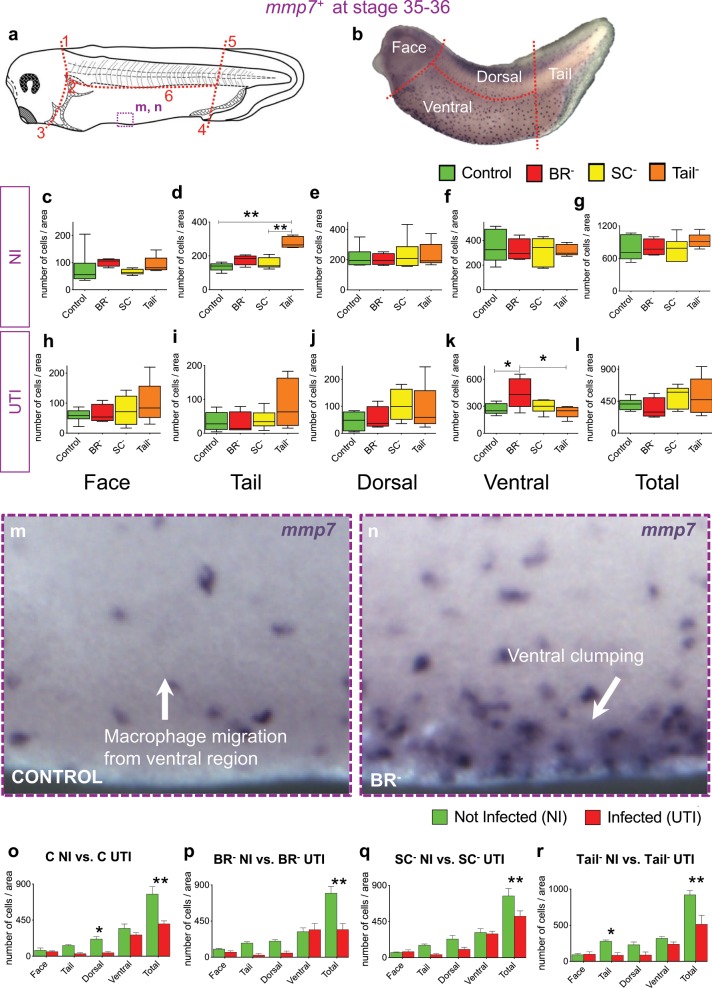
The absence of the brain during development affects the migration of macrophages in response to infection. **a**, **b** Drawing of (**a**) and image after in situ hybridization (ISH) for *mmp7*^*+*^ (**b**) of one st. 35 *Xenopus* embryo indicating the landmarks or reference points (1–6) for the body subdivision in the four independent areas for quantification (see Methods for details). Dashed-purple line square indicates the area shown in m, n inserts. Rostral is left and dorsal is up. **c**–**l** Quantification of number of *mmp7*^*+*^ cells in not-infected (NI, **c**–**g**) and infected (UTI, **h**–**l**), belonging each experimental group: Control (Ctrl, green), brainless (BR^–^; red), spinal cord resection (SC^–^; yellow) and Tailless (Tail^–^; orange). Values for normalized number of *mmp7*^*+*^ cells are plotted per each region and group: face (**c**, **h**), tail (**d**, **i**), dorsal (**e**, **j**), and ventral (**f**, **k**) to detect distribution and migration patterns. Total (**g**, **l**) is the sum of the four regions. One-way ANOVA *P* value showed significance for (**d**) (*P* *<* 0.01) and (**k**) (*P* *<* 0.05). **m**, **n** High-magnification images showing detail of ventral region (purple square in (**a**)) with *mmp7*-positive cells in a Control (**m**) vs. Brainless (**n**) embryos. VBI: Ventral Blood Islands or primitive hematopoietic organ. **o**–**r** Number of *mmp7*-positive myeloid cells (normalized to the area) in not-infected (green) vs. infected (red) embryos belonging Ctrl (**o**), BR^–^ (**p**), SC^–^ (**q**), and Tail^–^ (**r**) groups. Counts were done on four independent regions of the whole animal body (plus the summation): face, tail, dorsal and ventral areas. Two-way ANOVA showed significant *P* values for all comparisons (*P* < 0.05). **c**–**l**, **o**–**r** Data represent the mean and S.D. of, at least, ten different embryos, from three different replicates, per each group and condition. Significant *P* values after post hoc Bonferroni test are indicated as *P *<* 0.05 and **P *<* 0.01. See also Supplementary Fig. S2.

Considering that the early brain does not control the primitive formation of myeloid cells at early stages, we decided to evaluate the dynamic behavior or migration of immune cells at later stages. We labeled a gene coding for a protein expressed by *Xenopus* embryonic macrophages, *mmp7*, that mediates extracellular matrix remodeling, a crucial aspect of their migration.^11,48^ Homologs of this gene-protein have been extensively used to study macrophage behavior.^10,11,48^ *mmp7*-ISH was performed for the four experimental groups (Ctrl, BR^–^, SC^–^, Tail^–^) per each infection condition (UTI vs. NI) in st. 36 embryos. At least, ten different embryos, from different replicates, per each group and condition were used for quantification (*r* = 3, *n* = 40, *N* = 120; Fig. 3). In absence of infection (NI, Fig. 3c–g), the *mmp7*^+^ population was only significantly different at the injury site for Tail^–^ animals, as seen with the earlier myeloid *spiba*^*+*^ cells, with an increased number of mmp7^+^ cells accumulated in the bud tail or amputation plane respect to Ctrl embryos (one-way ANOVA *P* *<* 0.01; Bonferroni’s posttest *P* < 0.0001; Fig. 3d). No differences were detected for any area between Ctrl and BR^–^ animals, suggesting that the brain, in absence of infection, does not affect macrophage migration in the developing embryo. However, in embryos infected early in development with *E. coli* (UTI, Fig. 3h–l), absence of brain led to a significant increase in the number of *mmp7*^*+*^ cells in ventral areas respect to Ctrl animals (one-way ANOVA *P* *<* 0.05; Bonferroni’s posttest *P* = 0.0464 for BR^–^ vs. Ctrl at ventral area; Fig. 3k), represented by the accumulation of this population along the ventral hematopoietic niche (Fig. 3m, n). The ventral clumping of *mmp7*-positive cells in BR^–^ after infection was not detected in SC^–^ and Tail^–^ embryos, resulting in significant differences between BR^–^ and Tail^–^ groups (Bonferroni’s posttest *P* = 0.0225 for BR^–^ vs. Tail^–^ at ventral area; Fig. 3k), and demonstrating that the effect was not due simply to surgical damage or the need for an intact CNS.

The intragroup comparisons (Fig. 3o–r), analyzing embryos from the same surgery intervention but infected vs. not-infected conditions, and similarly to *spiba*^*+*^ population, demonstrated that the overall number of *mmp7*^+^ cells was lower in infected embryos, regardless of experimental intervention. This result confirms that the human uropathogenic *E. coli* targets the early innate immune system. Interestingly, the differences previously detected between Ctrl and Tail^–^ embryos in the tail area injury site, due to the tail injury itself, with a significant macrophage mobilization to the amputation plane (tail-bud area) observable only in absence of infection (Fig. 3d vs. Fig. 3i), were also displayed when comparing the effects of infection within the Tail^–^ group (Fig. 3r). A significant difference in the number of macrophages at the tail region or amputation site is detected between not-infected and UTI-infected Tail^−^ embryos (Bonferroni’s posttest *P* = 0.0487 for Tail^−^ NI vs. Tail^−^ UTI at tail region), indicating that the macrophage accumulation to the amputation plane in this group is entirely impaired in presence of bacterial infection. Our results show that the absence of the brain during development leads to defects in the response against infection, specifically the accumulation of *mmp7*^+^ cells in the ventral niche reveals defects in macrophage migration.

### Brainless induces ectopic myeloid and neural networks

Given the observed effects of the brain on the response of the developing innate immune (∼st. 30), we next analyzed effects of the brain’s presence on the fully-developed innate system^5,7,49–51^ and the surrounding innervation, in embryos at later stages of development (∼st. 44–48; see Supplementary Note 1 for more details about embryogenesis of the immune system in *Xenopus*).

**Fig. 4:**
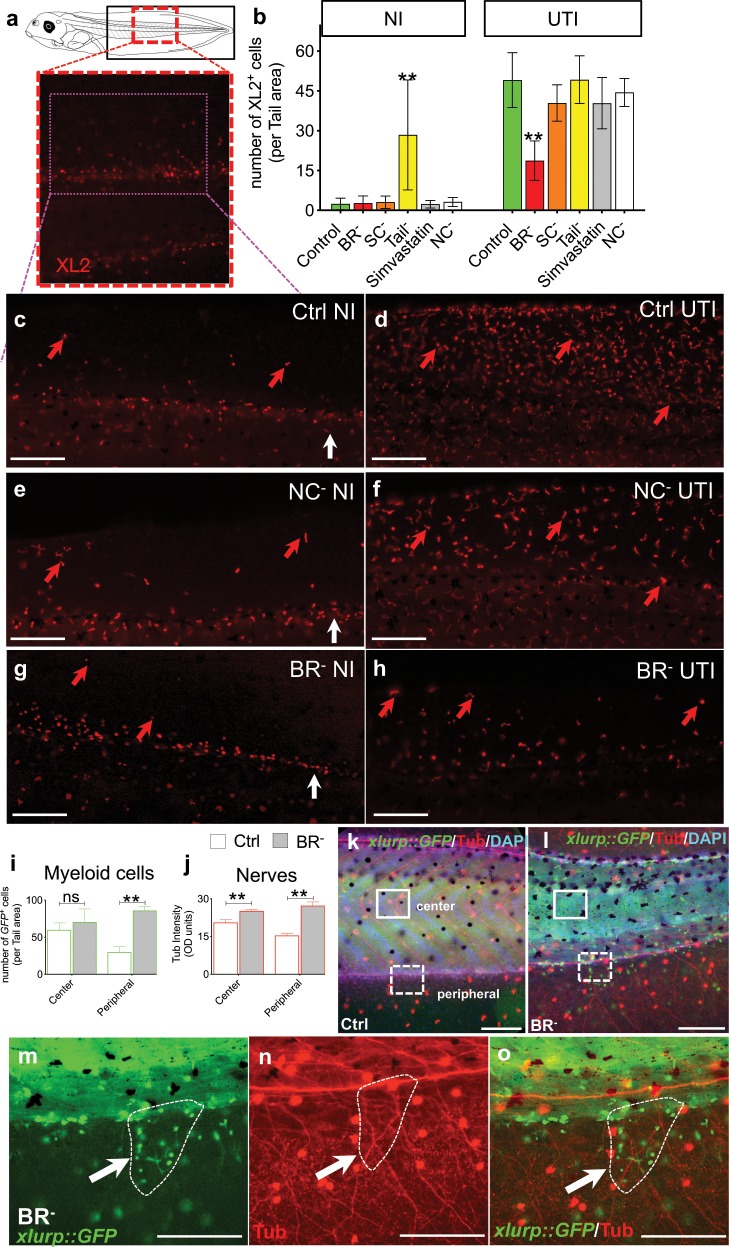
The absence of early brain affects number and patterning of myeloid cells, distributed in proximity to the aberrant neural network. **a**–**h** XL2 immunofluorescence to label leukocytes in uninfected (NI) and infected (UTI) embryos belonging Control (Ctrl, green), Brainless (BR^–^, red), Spinal Cord Resection (SC^–^), Tailless (Tail^–^), Simvastatin-treated, and Neural Crest ablation (NC^–^) groups. **a** Drawing of a late-staged *Xenopus* embryo. Black rectangle indicates the tail area for counting. Magenta-dashed line outlines the region showed in (**c**)–(**h**). **b** Number of XL2^+^ cells (normalized to the area) per each group and condition. **c**–**h** Lateral views of Ctrl (**c**, **d**), NC^–^ (**e**, **f**), and BR^–^ (**g**, **h**); NI (**c**, **e**, **g**) vs. UTI (**d**, **f**, **h**) embryos. Red arrows point XL2^+^ cells. White arrows point the posterior region of the central blood vessels. **i**, **j** Number of myeloid cells (**i**) and acetylated alpha-tubulin (Tub) optical density (OD; **j**) per each tail region for Ctrl (white), and BR^–^ (gray) st. 46–48 embryos. **k**–**o** Lateral views of *xlurp::GFP* Ctrl (**k**) and BR^–^ (**l**) embryos after Tub immunofluorescence. GFP^+^ myeloid cells are green, Tub^+^ nerves are red, nuclei are blue after DAPI staining. Differences in tail morphology for BR^–^ animals (deviating the normal body axis (**l**) with respect to Ctrl (**k**)) are due to developmental muscle mispatterning occurring when brain is absent. Two independent tail regions were counted: center (occupied by the myotomes; solid-white line) and peripheral (dorsal and ventral lateral fins; dashed-white line). Counts were made at the same region of the animals (e.g., at the same somite level). **m**–**o** Details from (**l**), white-dashed square. White arrows indicate positive elements at the same point on the three images. Dashed-white line circle an area within scattered groups of immune cells lying in proximity to the highly disorganized neural networks. **b**, **i**, **j** Data represent the mean and SD of, at least, ten different embryos, from different replicates, per each group and condition. Significant *P* values after post hoc tests are indicated as **P *<* 0.01, ns *P* > 0.05. Images: rostral is left, dorsal is up. Scale bar = 250 μm. See also Supplementary Fig. S3.

Immunofluorescence using the XL-2 antibody^8^ revealed the distribution of all *Xenopus* leukocytes (monoclonal antibodies specific for particular leukocyte subpopulations are not currently available for these species).^1^ We quantified XL2^+^ cells along the middle and posterior parts of the animal body in not-infected and infected st. 44–45 Ctrl, BR^–^, SC^–^, Tail^–^, and Simv embryos (Fig. 4a–h). In addition, we included the NC^–^ group in the XL2 analysis to study whether this intervention led to similar phenotypes of leukocyte migration than those ones expressed by BR^–^ animals (Fig. 4e, f). At least, ten different embryos, from different replicates, per each group and condition were used for quantification (*r* = 3, *n* = 40, *N* = 120). Our results for NI animals showed similar leukocyte patterns and number among all the groups, being tail amputation the only intervention that provoked a differential increase of XL2^+^ cells in the tail region (two-way ANOVA *P* *<* 0.01; Bonferroni’s posttest *P* *<* 0.0001 for Tail^–^ vs. the rest of the groups; Fig. 4b). Then, we analyzed the XL2 expression in UTI-infected animals subjected to the different interventions. Remarkably, the population of XL2-positive cells after infection for all groups was 2–4-fold greater than in absence of infection, with leukocytes leaving the central blood vessels and spreading along the entire fin. UTI-infected BR^–^ animals expressed the lowest XL2-positive population (19 ± 7 vs. 49 ± 10 XL2-positive cells per area in BR^–^ vs. Ctrl, respectively), with significant differences respect the rest of interventions (Bonferroni’s posttest *P* *<* 0.0001 for BR^–^ vs. the rest of the groups).

Thus, these results confirmed our observations at early stages of development. While in absence of bacterial threat, brain is not required for specifying leukocyte number, in presence of bacterial threat brain is required to induce an appropriate response-migrating behavior of the immune cells.

Next, we analyzed the transgenic *xlurp::GFP* animals, which express GFP in myeloid cells (mainly monocyte/macrophage and granulocyte/neutrophils at these stages)^5,52^ at st. 46–48 of Ctrl, BR^–^, SC^–^, Tail^–^ and Simv embryos. At least, ten different embryos, from different replicates, per each group and condition were used for quantification (*r* = 3, *n* = 40, *N* = 120; Fig. 4i–o, Supplementary Fig. S3, and Supplementary Video S1). One conspicuous feature observed in BR^–^ animals was the presence of longitudinal-like patches of high-density of GFP-positive cells along the dorsal and ventral fin, not detected in the somite or central regions of the body. Consequently, we decided to evaluate the number of immune cells for each experimental group on two independent tail regions: center and periphery, with somite region and fin region, respectively (Fig. 4i–l). The central region after tail removal (Tail^–^: 97 ± 27 GFP^+^ cells) exhibited a marked increase in the number of myeloid cells (normalized to area) compared with Ctrl embryos (59 ± 28 GFP^+^ cells; Dunn’s posttest *P* *=* 0.0373; Supplementary Fig. S3a, n), mainly focused at the region close to the amputation plane or injury site, indicating that even weeks after injury, myeloid cells are still invading the injured tail area. This effect was, conversely, not detected after brain removal, as more immune cells were not present either in the face region surrounding the injury site nor the central area. In the peripheral region, absence of the brain provoked a drastic increase in the number of myeloid cells at these late stages, forming defined patches that branched off the fin of the tail, at long distance from the injury site (from 30 ± 21 GFP^+^ cells in Ctrl group to 86 ± 15 GFP^+^ cells in BR^–^ group; two-way ANOVA *P* < 0.05; Bonferroni’s posttest *P* < 0.0001). This sprouted-network like patterning of the myeloid population in the fin region of BR^–^ animals displayed a similar pattern to the one detected previously for the peripheral neural network in absence of brain.^36^

Thus, we decided to study the co-location of peripheral nerves with respect to this myeloid population (using Tub immunofluorescence on *xlurp::GFP* embryos). Morphological analysis revealed that, in central regions, myeloid cells of BR^–^ exhibited different morphology, with respect to other areas or to Ctrl embryos, with rows of flattened cells, compatible with a macrophage-like network,^5^ following the internal neuropil.^36^ This distribution pattern, with myeloid cells in close proximity to the internal neuropil, was entirely absent in Ctrl embryos, where the central areas, with the highest number of GFP cells (Fig. 4i, j; Supplementary Fig. S3e–g) were not intensely occupied by nerves. In the peripheral region, a similar distribution pattern for both myeloid cells and peripheral nerves was detected in BR^–^, with scattered groups of immune cells in proximity to the highly disorganized neural networks (see the area circled within the dashed-white line as a illustrative example in Fig. 4m–o; Supplementary Fig. S3k–m for Ctrl). SC^–^ animals exhibited a similar number and general distribution of myeloid cells as the Ctrl group but the peripheral-nerve phenotype, as revealed by Tub immunofluorescence, showed the same ectopic growth and sprouting of neural network as in the BR^–^ animals and significantly different from the Ctrl group (Supplementary Fig. S3o–q). Simv-treated embryos did not show differences for any marker, myeloid and nerve phenotypes, with respect to the Ctrl group.

Taken together, our results confirmed that brain removal during early development leads to the ectopic presence of mature myeloid cells at later stages of embryogenesis invading the fin, in close proximity to the aberrant peripheral neural network. This aberrant distribution of immune cells occurs far from the distal anterior injury site and is not mediated by spinal cord pathway. Lack of brain thus produces an immunologically different phenotype than other interventions, such as the removal of a different piece of the body (which induces the increased presence of immune cells but only at the local area of injury), or a severe toxic stress (Simv treatment, which caused no differences in myeloid cells relative to controls), indicating the unique influence of the embryonic brain on immune cell behavior.

### Brain controls the transcriptional consequences of infection

**Fig. 5:**
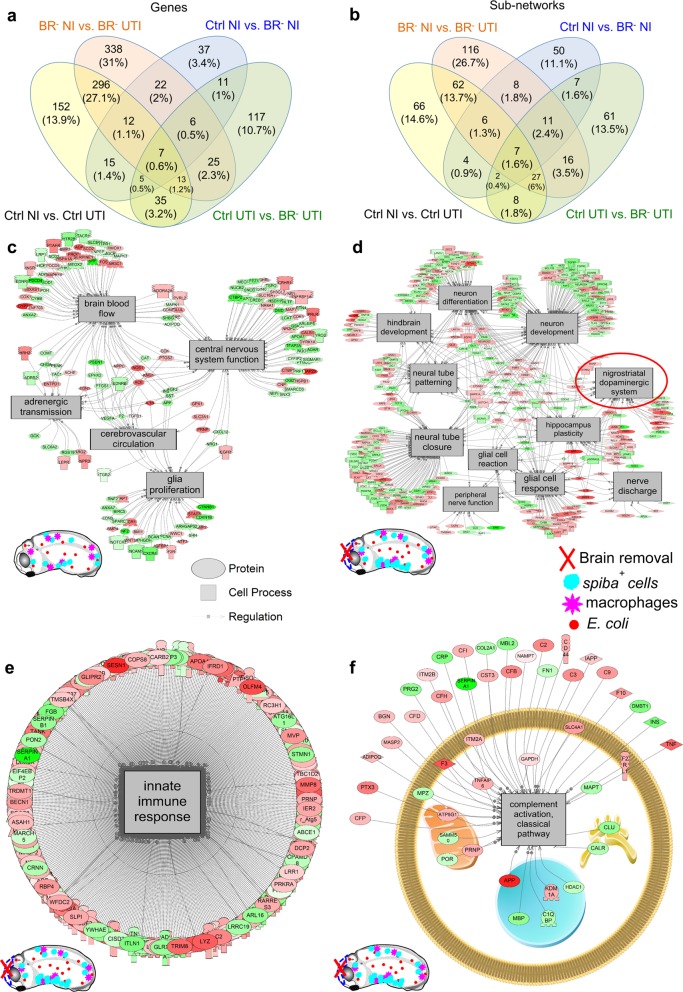
Transcriptional analysis of Control (Ctrl) and Brainless (BR^–^) datasets show quantitatively and qualitatively differences for regulated transcripts and cell processes after infection (UTI) or/and not-infection (NI) conditions. **a**, **b** Venn diagram comparing genes (**a**) and sub-networks (**b**) differentially regulated for each experimental group-condition. **c** Neural-related pathways unique to infection with a brain. **d** Neural-related pathways unique to infection without a brain. **e** Innate immunity response for brainless animals with infection. This network was significantly upregulated by 11%. **f** Complement activation sub-network (classical pathway) exclusively present in infected embryos with absence of brain. Green = down gene, Red = up gene. Complete data are presented in Supplementary Data 1. All measured genes found in a pathway are located in Supplementary Data 2. See also Supplementary Figs S4 and S5.

Given the differences found for the innate immune response with or without a brain, we sought to identify the transcriptional mechanisms underlying the effects. To characterize the transcripts that could be differentially regulated under each condition, we conducted RNA-seq and compared the transcriptome of Ctrl and BR^–^ embryos, with or without infection (Fig. 5, Supplementary Tables S1–S7, Supplementary Figs. S4 and S5, Supplementary Note 2, and Supplementary Data 1 and 2).

We first asked which transcripts are controlled by infection in an intact normal embryo by comparing the transcriptome of NI vs. UTI Control animals (Ctrl NI vs. Ctrl UTI, black labels in Fig. 5a). A total of 535 DEGs were regulated by infection in intact embryos, and 152 were unique to this group. Conversely, this number was significantly increased up to 719 (and 338 unique) transcripts controlled by infection in brainless embryos (or BR^–^ NI vs. BR^–^ infected; orange labels in Fig. 5a), revealing that the presence of the brain suppresses the transcriptional consequences of infection about twofold. In the absence of infection (comparing Ctrl NI vs. BR^–^ NI, blue labels in Fig. 5a), we found that 115 genes (and 37 unique) were responsive to brain removal per se. To characterize the transcriptional response specifically due to brain removal, and not to the removal of any organ in general, we compared the dataset of DEGs after brain removal from our study with the DEGs obtained after tail removal that were identified in two prior studies, in *Xenopus*^53^ and lizard.^54^ The tail is large appendage including a massive CNS component (spinal cord) and triggers a robust regenerative response, allowing us to identify and exclude from our analysis genes that are not specific to the removal of brain. After subtracting the common transcripts between brain and tail removal, we found that, from the initial 115 genes, 91 were unique to our dataset for brain removal and 24 were common with tail amputation (for the complete list of genes, see Supplementary Data 1, Brain Removal vs. Tail Removal). These data indicate that about 75% of the transcripts detected in our study after brain removal are unique to this intervention. The last comparison, Ctrl UTI vs. BR^–^ UTI (green labels in Fig. 5a) revealed that the response to infection in the absence of brain affects the transcription of 218 (117 unique) genes.

Next, we extracted the specific genes with both the highest response (up- or downregulation) and unique (exclusively expressed) within each comparison (see Supplementary Data 1 for the complete list of genes; Supplementary Tables S2–S8 and Supplementary Note 2 for *Summary of the top differentially expressed genes*), concluding that most of the genes implicated in the bidirectional communication brain-infection belong to TNFR-I signaling pathway and encoding of ligands and receptors specific of immune/myeloid cells. Next, we grouped the differentially enriched elements according to their “large-scale functions” to reveal the types of processes mainly regulated within each brain-infection condition. The more predominant gene networks were related to blood, bone, ion transport or transmembrane potential (bioelectric signaling),^55,56^ apoptosis, development and regeneration, neural, immune response and bacteria (Supplementary Fig. S4a–d). The bioelectric-related genes (ion transport) occupied 3% of the total DEGs in control embryos under infection (Supplementary Fig. S4a), while this percentage was clearly lower in the rest of groups (around 1%), consistent with the known role of bioelectric signaling in the innate immune response.^9^ The removal of the brain (in absence of infection) did not induce any changes in the transcription of genes related to bone (0%), with the neural-related functions the most affected (10% of the total of the transcriptome for Ctrl NI vs. BR^–^ NI; Supplementary Fig. S4c). How the absence of a brain affects the response to infection (Ctrl UTI vs. BR^–^ UTI; Supplementary Fig. S4d) was reflected by considerable changes (respect to Ctrl NI vs. Ctrl UTI) in the percentages of immune-related transcripts (−8%), neural-related (+6%), bone (−4%), and developmental and regeneration-related (+2%) functions.

To reveal the gene regulatory networks (GRN) and motifs and features that were statistically overrepresented, we performed SNEA (Fig. 5c–f, Supplementary Figs S4 and S5, and Supplementary Data 1 and 2 for the complete list of pathways). In animals developed with a brain, 80 pathways were controlled by the infection (66 of them were unique to Ctrl NI vs. Ctrl UTI; black labels in Fig. 5b). Conversely, in animals developed without a brain (RNA isolation took place after brain removal), infection induced the differential regulation of 151 pathways (116 were unique to BR^–^ NI vs. BR^–^ UTI; orange labels in Fig. 5b), suggesting that about 70% of the response to infection depends on the presence of brain.

In grouping the neural-related pathways, we determined that three elements were shared by Ctrl UTI and BR^–^ UTI (innervation, neurogenesis and brain microcirculation), five sub-networks were included exclusively in infected embryos with brain (Ctrl UTI: adrenergic transmission, CNS function, glia proliferation, brain blood flow and cerebrovascular circulation; Fig. 5c) and eleven were unique to infected embryos without brain (BR^–^ UTI: dopaminergic system, glial cell reaction, glial cell response, hindbrain development, hippocampus plasticity, nerve discharge, neural tube closure, neural tube patterning, neuron development, neuron differentiation, and peripheral-nerve function; Fig. 5d). Intriguingly, in the latter category, networks related to bacteria were upregulated by 20%, overall as a group (Supplementary Fig. S4e). The only common pathways regulated in both response to brain removal and tail removal were microtubule- and cell division-related (cell cycle regulation, cell fate, chromatin remodeling, chromosome movement, epithelium development, microtubule bundling, microtubule cytoskeleton organization, microtubule sliding, and microtubule/kinetochore interaction). Some of the unique pathways differentially regulated after brain removal only, include apoptosis of neutrophils, apoptotic chromosome condensation, DNA annealing, DNA damage excision, DNA damage recognition, DNA end joining repair, DNA strand breakage, neurogenesis, neuron development, and neuron differentiation. The sub-network related to innate immune response was one of the most affected by the presence/absence of brain. Specifically, the innate immunity for brainless embryos with infection exhibited an upregulation of up to 11% (median change in the network of 1.11-fold; Fig. 5e). Some of these unique immune-pathways regulated in absence of brain were complement activation (classical pathway, Fig. 5f), immune complex clearance and activation, macrophage-focused (adhesion, apoptosis, fusion) and neutrophil-focused (activation, chemotaxis, extravasation and recruitment; all genes within a differentially expressed pathway are provided in Supplementary Data 2).

Thus, our analysis revealed profound changes in the transcriptional networks responsive to infection induced by absence of brain, suggesting a key role of the early brain in producing a robust innate immune response in response to systemic infection and implicating a variety of factors related to immune and neural pathways such as migration or dopaminergic transmission.

### Dopamine is implied in the protective effects of the brain

Considering the molecular candidates and pathways revealed by RNA-seq, we next targeted one of the regulatory networks exclusively affected after infection in the absence of the brain. From the 11 neural pathways unique to BR^–^ UTI, we decide to validate the possible role of the dopaminergic signaling in mediating the increased susceptibility to infection, as it has been also implicated in macrophage migration.^57^

**Fig. 6:**
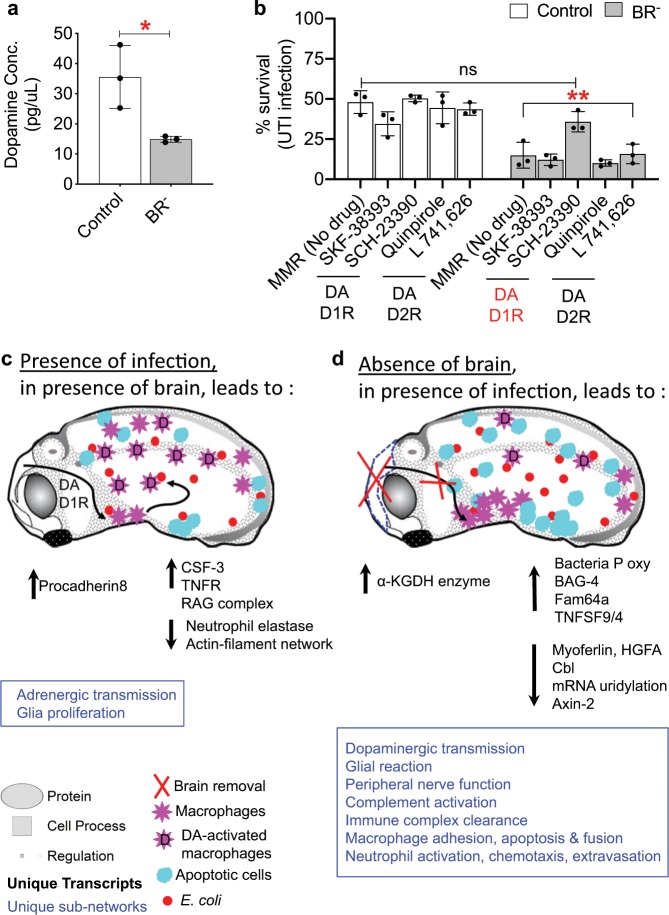
Functional assays demonstrate a role for dopamine signaling in the protective effects of the brain on immune response. **a** Dopamine concentration (in pg/μL) in infected Control (Ctrl, white) and Brainless (BR^–^, gray) embryos at early stage 35, measured by liquid chromatography–mass spectrometry (LC–MS/MS). Unpaired *t*-test *P < 0.05. **b** Survival rates (in percentage) of Ctrl (white) and BR^–^ (gray) infected embryos after pharmacological treatment with drugs targeting the type-1 or type-2 family of dopamine (DA) receptors (D1R, D2R). Two-way ANOVA *P* < 0.01; *P* values after Bonferroni post hoc test are indicated as **P *<* 0.01, ns *P* > 0.05. **a**, **b** Data represent the mean and S.D. of three independent replicates of 30 embryos. Each replicate is shown by one dot. See also Supplementary Fig. S6. **c**, **d** Drawings represent the morphological and molecular events occurs in response to infection, in presence (**c**) or absence (**d**) of brain. Unique neural- and immune-related pathways are indicated in blue. Upregulation and downregulation of transcripts are indicated in black. *E. coli* is represented by red circles; apoptotic cells are represented by cyan circles; *mmp7*^+^ cells (early macrophages) are represented by pink stars; dopamine-activated macrophages are represented with a D on the pink star. Complete list of genes is presented in Supplementary Data 1. Green = down gene, Red = up gene. All measured genes found in a pathway are located in Supplementary Data 2.

First, we functionally tested whether the DA levels were quantitatively different between Ctrl^−^ and BR^–^ infected embryos. Embryos were analyzed by LC–MS/MS at early st. 35 (~20 h post-surgery, 44 h post infection), immediately before the peak of infection-induced death in BR^–^ animals and the significant differences both in survival rate and apoptosis are reached respect to Ctrl embryos (Fig. 2a, b). DA turnover rates were significantly different between Ctrl (35.56 ± 10.49 pg/uL) and BR^–^ (14.90 ± 0.99 pg/μL) embryos (*r* = 3, *n* = 30; unpaired *t*-test *P* = 0.0274; Fig. 6a, Supplementary Fig. S6), confirming that the DA concentration in the tail region of the embryo after infection is differentially affected in the presence vs. absence of the brain.

Next, we performed pharmacological assays targeting the type-1 or type-2 family of dopamine (DA) receptors (D1R, D2R). We used specific agonists and antagonists of each dopamine receptor,^58^ such as the following: for D1R,^57,59^ SKF-38393^60^ (SKF) and SCH-23390^61^ (SCH) were used as an agonist and antagonist, respectively; for D2R, Quinpirole^57^ (Quin) and L-741,626^62^ (L741) were used as an agonist and antagonist, respectively. In order to understand whether we could mimic the BR^–^ phenotype (decreased survival rate) in Ctrl embryos and/or mimic the Ctrl phenotype (increased survival rate) in BR^–^ embryos, we exposed Ctrl and BR^–^ infected embryos to the dopaminergic drugs from st. 25 (immediately after brain removal) to st. 48, where we evaluated the survival rate after each treatment (*r* = 3, *n* = 30, *N* = 90 embryos per treatment; Fig. 6b). For Ctrl infected embryos, no significant differences in the survival rates were found after exposure to any of the drugs. For BR^–^ infected embryos, exposure to SCH (D1R antagonist) rescued embryos from the increased death (14.8 ± 8% with no drug) to a survival rate of 36 ± 6%, displaying significant differences respect to the values reached for the rest of no-drug/drug-treated BR^–^ embryos (two-way ANOVA *P* < 0.01; Bonferroni’s posttest for BR^–^: SCH vs. MMR *P* = 0.0049; SCH vs. SKF *P* = 0.0015; SCH vs. Quin *P* = 0.0006; SCH vs. L741 *P* = 0.0071) and no differences respect to no-drug Ctrl embryos (48 ± 7%, Bonferroni’s posttest *P* = 0.1878 for BR^–^ SCH vs. Ctrl. MMR). These results confirmed, using functional assays, that the dopaminergic regulatory network is affected in BR^–^ infected embryos, and that antagonism of the D1R signaling pathway can rescue the induced-infection death detected in absence of brain, mimicking the protective effects of the brain signals on the immune response to infection.

## Discussion

Immunology, developmental biology, neuroscience, and regenerative medicine are all converging in an emerging interdisciplinary field of profound significance for biomedicine as well as basic biology.^63^ While the brain–immune axis is now beginning to be characterized,^9,17,64^ many gaps exist in our understanding of the functional links between the brain, response to infection, and regenerative processes triggered by wound healing.^65^ This is especially true for the earliest developmental events that shape the future interactions of these subsystems during health and disease. Here, we exploited the highly tractable *Xenopus laevis* model to identify unrevealed aspects of the innate immune response that rely on the presence of brain, and we characterized the cell-level and transcriptional machinery that underlies the effects.

We challenged *Xenopus* embryos with UTI *E. coli* load of 4.5 × 10^3^ cfu/ml; this dose is up to five orders of magnitude smaller than some model organisms and human organs tolerate,^66–69^ providing a comfortable dynamic range within which to evaluate susceptibility and tolerance. Crucially, we show that the specific presence of the brain strongly impacts the ability of embryos to survive bacterial challenge (Fig. 1). The early brain is protecting the embryo by inducing cellular and physiological responses (decreasing the infection-induced damage and apoptosis; Fig. 2, and promoting macrophage migration; Figs 3 and 4) and molecular mechanisms (suppressing transcriptional consequences of the infection; Fig. 5) that help to overcome the bacterial threat. Pharmacological and functional assays revealed that absence of brain during infection leads to decreased levels of peripheral DA. Targeting the DA-D1R signaling pathway in brainless animals rescues them from infection-induced death (Fig. 6). These results suggest that brain-derived DA signaling is key in mediating the protective effects of the brain signals in response to infection.

The brain is not necessary for the early development and proliferation of primitive myeloid precursors (Supplementary Fig. S2). However, in response to infection, signals from the brain are required for proper macrophage migration (Fig. 3), revealing that brain-dependent signals regulate the behavior of immune cells. Without infection, at later stages with a more mature immune system, the absence of a brain leads to aberrant and ectopic distribution of macrophages and neutrophils (Fig. 4), which occurs in the context of an ectopic neural network which could be a factor guiding the abnormal distribution of immune cells (Fig. 4m–o). Importantly, removing the brain does not induce the migration of immune cells to the injury site (the head region), which clearly occurs with other parts removed from the body, such as tail amputation (Supplementary Fig. S3n).

The protective effects of the brain do not require spinal cord contiguity, an important fact for the design of immune-enhancing therapies. Survival rate of SC^–^ embryos after infection is similar to Ctrl (Fig. 1i), and mature immune cells in SC^–^ animals have similar numbers and general distribution as the control group (Supplementary Fig. S3a, b). Conversely, the peripheral-nerve phenotype in SC^–^ animals showed the same ectopic growth and sprouting of neural network than in BR^–^ animals (Supplementary Fig. S3o–q). Thus, unlike the peripheral-nerve distribution, immune cell behavior is not controlled via the spinal cord. Likewise, neural crest ablation did not recapitulate effects of brain removal (Supplementary Fig. S1e–g), consistent with the essential role of the brain per se.

Important information was provided by two additional groups: tailless (a control for general surgical damage), and the Simvastatin-treated group (Simv, a control for general tissue stress). Simvastatin causes severe myotoxic effects in humans and zebrafish embryos,^39,40^ leading to death from continuous exposure or above μM concentration, indicative of a strong drug-derived stress. In *Xenopus* embryos, Simv treatment led to severe aberrant muscle phenotypes, without altering the brain morphology (Supplementary Fig. S1h–j). Survival after infection (Fig. 1f) and the immune phenotype (Supplementary Fig. S3) of the Simv group are not significantly different than the Ctrl group, indicating that muscle alterations are not a primary cause of immune defects, and the combination of infection + strong stressor does not reduce survival. Thus, the absence of brain when a bacterial infection is present leads to dysfunctional macrophage behavior and high susceptibility to infection that is not recapitulated by even severe general stressors or tissue damage.

To more fully understand the link between brain and immunity, we assessed the bacterial load at 48 h after infection and the relation between survival and pathogen load, using a metric that combines these two variables in a single number: Host-Pathogen Response Index (HPRI; Fig. 1i). Comparing survival percentage across the control infected vs. brainless infected vs. brainless noninfected (Fig. 1f, g) under constant pathogen load confirmed brain-dependent susceptibility, which includes a tolerance component (Fig. 1h, i). In addition to the brain, it is likely that other components of the circuit could be discovered in the future. The cellular and morphological mechanisms behind the higher susceptibility in absence of the brain include increased apoptosis and inflammation induced by infection (Fig. 2), which promote an earlier peak of death and hamper the recovery from infection and normal development to st. 48.

Analysis of the embryos’ robust transcriptional response shed light on the pathways related to brain → body, bacteria → body, and bacteria → brainless body signaling. Infection when brain is present induces the differential expression of immune-related elements such as antigen recognition, leukocyte cell adhesion, lymphoid differentiation, T-cell proliferative response and tolerance, basophil activation, disease resistance, thrombocyte aggregation, and virus morphogenesis. Infection. Similarly, some of the more significantly upregulated genes, include genes encoding proteins (ligand-receptor) for immune cell functioning (Fig. 5, Supplementary Fig. S5), such as the gene encoding colony-stimulating factor 3 (CSF 3, a granulocyte growth factor necessary for the differentiation of bone marrow cells to granulocyte-lineage^70^), the gene encoding for receptor-interacting serine/threonine-protein kinase 3-like (RIP, a component of the Tumor Necrosis Factor-receptor I (TNF-R1) signaling complex,^71^ and RAG complex genes involved in maturation of the antibody repertoire of adaptive immunity. The differential regulation of the TNF-signaling pathway is consistent with recent mammalian data showing that innate immune response in mice to *Listeria monocytogenes* results in upregulation of TNF in cerebrovascular fluid.^2^

The deficient response to infection detected in brainless animals, with low survival rates and defects in immune cell location, was transcriptionally reflected, with an increased activity of the innate immune sub-network by ~11–12% (Fig. 5e) compared with animals with a brain. The ineffective upregulation of transcripts may indicate compensatory transcriptional responses in the body from other systems in the absence of a brain when challenged with a pathogen.^72^ RNA-seq revealed the most significant immune and neural pathways and transcripts affected by absence of brain such as complement activation, macrophage-focused (adhesion, apoptosis, fusion), neutrophil-focused (activation, chemotaxis, extravasation and recruitment), or overexpression of BAG-4 related genes (or TNF-R1 silencers) and cell adhesion proteins (VCAM-1). Networks functionally related to bacteria are inhibited by 20% and eleven neural pathways are uniquely affected after infection when brain is not present.

The dopaminergic transmission is one of the most affected regulatory networks in presence of infection when the brain is absent, and levels of peripheral DA are decreased in absence of brain. Macrophage migration is one of the targets for the protective role of the brain and an increasing number of recent studies relate dopamine and inflammation and immunity.^57,73,74^ Based on our cellular, molecular and pharmacological results, a possible mechanism explaining the protective effects of the brain in presence of bacteria is illustrated in the drawings-models of Fig. 6c, d. In presence of bacteria, the immune response of the embryo is initiated in the early brain (st. 25) and communicated to the periphery by modulating DA-signaling pathways in macrophages (trough D1R antagonism) during the first 48 h post infection (or before st. 35). The DA-activated macrophages migrate and act systemically decreasing apoptosis and infection-induced inflammation. As a consequence, embryo’s tolerance to infection is harnessed and by st. 40, induced-infection death is entirely stabilized. In absence of brain, low levels of peripheral DA cannot activate immune cells and promote them to initiate the migratory response. Consequently, in absence of a macrophage network, brainless embryos become more susceptible to the lethal effects of the bacterial infection, which induces high levels of inflammation and apoptotic events that lead to an earlier and massive peak of death by st. 35.

Brainless animals showed an aberrant overexpression of central- (nigrostriatal dopamine) and peripheral- (peripheral-nerve function) neural networks (Fig. 5d), as well as bacteria-related pathways (Fig. S4e), revealing responses of the host to microbial presence that could explain the differences in the HPRI and, consequently, the lower survival rates detected in brainless animals after infection. Specifically, the TNF-R1 pathway is significantly upregulated in intact embryos (developed with brain) in presence of infection, and it seems to be deregulated when brain is absent. Brainless infected animals overexpress genes related to BAG-4 or silencer of death domains (SODD)—a widely expressed 60-kiloDalton protein that associate with the death domain of TNF-R1, silencing it when overexpression is detected.^75^ The accumulation of immune cells in niche in absence of brain (Fig. 3m, n) is also detected in transcriptome changes, with the significant upregulation of the vascular cell adhesion protein 1 (VCAM-1) that mediates the adhesion of immune cells. Cell cycle and chromatin-expression regulators, such as ubiquitination of proteins (mediated by tnip1 or cbl), methylation (via downregulation of euchromatic histone-lysine N-methyltransferase 1L that methylates the lysine-9 position of histone H3 and tags it for transcriptional repression) or uridylation of mRNAs appear significantly and exclusively regulated in brainless animals (with or without infection). In infected animals, absence of brain induces the specific networks for ubiquitization of CSF-1R (macrophage receptor for growth and proliferation). This mechanism, along with high expression of adhesion proteins, could be the responsible for the attenuation in the macrophage proliferation detected in brainless animals.

In conclusion, a unique vertebrate model that develops without a brain enabled morphological, transcriptional and functional evidence for brain-mediated modulation of the immune system reactivity. Specifically, we demonstrate that the absence of brain makes embryos more susceptible to pathogen, lowering tolerance^41,42^ and increasing apoptosis and inflammation. The influence of the endogenous brain seems to be mediated by control of cell localization, especially affecting macrophage migration to fight infection. RNA-seq revealed the most significant immune and neural pathways and transcripts affected by absence of brain. Overall, 70% of the response at the cellular network level is different based on the presence/absence of brain. Our functional assays reveal that DA signaling could be key in the bacteria–brain–immune crosstalk, suggesting that modulation of dopamine receptors could be an important strategy for mimicking the protective effects of brain signals on the immune response to infection in biomedical settings.

The brain is an active component of the innate immune response. Future work will focus on decoding the bioelectric and biochemical signals that mediate its effects on distant immune cells and identifying heretofore unrecognized cellular targets of these signals. More broadly, beyond the brain–body–bacteria axis, the understanding of the influence of the brain over cell- and molecular-level processes is an interesting frontier, with numerous potential applications across basic biology and medicine. A full understanding of brain effects on cellular behavior (complementing neuroscientists’ focus on whole animal behaviors) is likely to not only shed light on the evolution of neural and immune systems but also to facilitate the development of intervention strategies in biomedical settings. We speculate that combinations of appropriate bioelectrical and neurotransmitter signals can become a useful tool for addressing infectious and other disease states.

## Methods

### Animal husbandry

*Xenopus laevis* wild-type and transgenic embryos were fertilized in vitro according to standard protocols^76^ in 0.1X Marc’s Modified Ringer’s solution (MMR; 10 mM Na^+^, 0.2 mM K^+^, 10.5 mM Cl^–^, 0.2 mM Ca^2+^, pH 7.8). The transgenic *Xenopus*, expressing Green Fluorescent Protein (GFP) under the control of the *lurp1* gene promoter, *xlurp::GFP*, was obtained from the Marine Biological Laboratory (MBL, National Xenopus Resource RRID:SCR_013731).^77^ *Xenopus* embryos were housed at 18–21 °C and staged according to Nieuwkoop and Faber.^78^ All experimental procedures involving *Xenopus* embryos were approved by the Institutional Animal Care and Use Committees and Tufts University Department of Laboratory Animal Medicine under protocol M2017-53.

### Bacterial injections, survival and host-pathogen assays

All bacterial strains were generously provided by Dr. Matthew A. Mulvey (University of Utah, Salt Lake City, Utah, USA) and have been previously described.^79^ We utilized an uropathogenic *Escherichia coli* (*E. coli*) strain UTI89 to elicit a pathogenic response. Bacteria were grown at 37 °C in LB medium (MP Biomedicals, LLC) supplemented with 50 mg/mL ampicillin (Fisher). One milliliter of the UTI89 stock (1 × 10^9^ colony forming units per ml (cfu/ml)) overnight bacterial culture was pelleted and resuspended to a stock concentration of 1 × 10^10^ cfu/mL in phosphate-buffered saline before injections in embryos. Gastrula stage embryos were injected using borosilicate glass needles calibrated for a bubble pressure of 25–30 kDa and 150 ms pulses. All known variables were kept consistent across biological and technical replicates. To calculate the injected cfu, embryos from different biological replicates were harvested immediately after injection (*t* = 0 h) for subsequent lysis and plating. Later, embryos infected with *E. coli* UTI 89 (UTI condition) and not-infected (NI condition) were incubated at 21 °C (Fig. 1a for general diagram of experimental design). Surgeries were performed 18–24 h after infection (st. 25). Survival within each experimental group was evaluated at several stages until embryos reached st. 46–48, or 4–5 d after infection. Even though bacteria were grown and concentrated following a constant methodology, and embryos were collected and grown under the same conditions, variation in survival rates was observed between experiments. This is likely due to differences in bacterial growth in situ and in the genetic background of each individual from different egg clutches. For these reasons, every individual comparison within an experiment was conducted using eggs from a single fertilization and a single suspension of bacteria, and controls were used within each study. At least 120 embryos were used for every treatment being compared, and each assay was tested in triplicate. Three additional injection assays were performed in order to calculate the number of bacteria per embryo at 48 h after infection. Bacterial load was determined after lysis of live embryos (five independent embryos/experimental group; TissueLyser for 30 s at 30 Hz) and following standard protocols of microbial plating analysis. Timing selection was established following previous published work,^9^ which demonstrated that the lethal effect of UTI89 infection on *Xenopus* embryos can be evaluated at 72 h and the survival rates maintain constant after 5 d. The Host-Pathogen Response Index (HPRI = % survival/(1 + log10(CFU + 1)) was developed to compare embryo survival with respect to pathogen load in order to provide a quantitative metric of susceptibility and tolerance that comprises both pathogen load and survival. HPRI scores of 0 indicating complete susceptibility and 100 indicating complete embryo survival and clearance of pathogen. The slope of HPRI over time or following a perturbation, reflects tolerance phenotypes.

### Experimental groups and microsurgery

At 18–24 h post infection, st. 25 infected (UTI condition) and not-infected (NI condition) embryos were randomly allocated in one of the four experimental groups, respectively (Fig. 1b–e, Supplementary Fig. S1): Control (Ctrl; Fig. 1b, c), Brainless (BR^–^; Fig. 1d, e), Spinal Cord resection (SC^–^; Supplementary Fig. S1c, d) or Tail-bud resection (Tail^–^). The SC^–^ group was used to test whether the effects are mediated by neural communication going through the spinal cord. We used Tailless embryos as control for invasiveness of removal of large amounts of tissue and as a positive control, since we have previously demonstrated this assay increases resistance to infection.^9^ Brain removal was performed under dissecting microscope, using a dissecting knife (FST #10055-12), via a single cut of the antero-dorsal region corresponding to the three main subdivisons of the developmental brain (see Supplementary Fig. S1a, b for images of stage-48 embryos showing that no brain regeneration takes place after removal at st. 25). For SC^–^, a consistent piece of the most cervical levels, sizing 50–100 μm in length, was completely removed by using two forceps with super-fine tips (Dumont #5ST, FST 11252-00). Tail buds were amputated from the most posterior fifth portion of their body using a scalpel blade (additional details of microsurgeries can be found in ref. ^36^). As an additional experimental control for the brain ablation, we included the group Neural Crest ablation (NC^–^; Supplementary Fig. S1e–g). Published protocols for ablation of the migrating cranial NC in Xenopus^80^ were followed at st. 17 embryos, ~6–8 h after infection.

After surgery, animals were allowed to heal in 0.75X MMR for 1 h, then raised in regular 0.1X MMR at 21 °C and scored and analyzed for percentage of survival rate at st. 26–27 (2–4 hps), st. 30 (8 hps), st. 35 (24 hps), st. 40 (36 hps), st. 42 (48 hps), and st. 46–48 (4 days ps). At each time point, embryos were harvested and prepared for morphological analysis (in situ hybridization or immunofluorescence). Molecular assays, RNA extraction and liquid chromatography-coupled tandem mass spectrometry (LC–MS/MS), were performed on whole embryos harvested at 3 and 20 hps, respectively.

Stage-25 UTI and NI *Xenopus* embryos were treated with 0.4 μM Simvastatin (Supplementary Fig. S1h–j; Tocris Cat. No. 1965/50) for 18 h at 22 °C. After 18-h exposure, embryos were transferred to fresh normal media (0.1X MMR) and allowed to develop up to the same stages as the rest of the groups for harvesting and posterior analysis. Stock solution of the drug was created by dissolving the compound in ethanol to a final concentration of 0.24 mM. Further dilution was made in 0.1X MMR.

### In situ hybridization and cell counting

In situ hybridization was performed as previously described^9^ on UTI and NI embryos belonging each experimental group: Ctrl, BR^–^, SC^–^, and Tail^–^. Briefly, specimens were washed in 0.1% Tween-20/phosphate-buffered saline (PBS-T) and dehydrated through increasing concentrations of methanol. *Xenopus laevis spib-a* (*spiba*; GE Dharmacon) and *Xenopus tropicalis mmp7* (matrix metalloproteinase-7) were used as probes and kindly provided by Enrique Amaya (University of Manchester). *spib-* is an ETS transcription factor, highly conserved with mammals,^10,47,81^ necessary for myeloid specification. *spiba* expression at st. 28 embryos marks the earliest primitive myeloid population.^3,10^ mmp7 is a secreted metalloproteinase involved in extracellular matrix remodeling and extensively used as macrophage differentiation and migration markers.^3,10,11,48,82^ *mmp7* probe was used to label migrating embryonic macrophages on st. 35–36 embryos, as relative to other myeloid markers, mmp7 is expressed considerably later.^10^ Probes were generated in vitro from linearized templates using a digoxigenin (DIG)-labeling mix (Sigma-Aldrich, #11277065910 Roche).

Quantification of *spiba-* (Supplementary Fig. S2) and *mmp7-* (Fig. 3) positive cells was performed using ImageJ software on bright-field images of whole-mount ISH st. 28 and st. 35 embryos, respectively. Images and counts were performed on the left side of the animals belonging each experimental group (Ctrl, BR^–^, SC^–^, Tail^–^) and for each condition (NI vs. UTI). Firstly, each image was carefully subdivided and cropped (to avoid overlapping) in four independent (face, tail, dorsal and ventral) using six reference points or landmarks (Fig. 3a, b, Supplementary Fig. S2a, b, s–w). Clearly identifiable landmarks for *Xenopus* embryos were orderly used (from 1 to 6) to decrease variability and bias during the blind counting, such as the following: (1) beginning of spinal cord (SC), most anterior portion or cervical levels, (2) intersection between posterior edge of the IV branchial arch (ba) and ventral edge for the first and most anterior somite (Sm); (3) anterior to the heart (h); (4) end of hindgut (hg); (5) beginning of the tail bud (tb); (6) ventral line delimited by somites. The face region is composed of the entire anterior area including the olfactory bulb, the eye area, and the otic vesicle, the tail region is composed of the posterior area including tail fin (posterior to the hindgut); the dorsal region is composed of the spinal cord, notochord, and somites (it begins directly posterior to the face region and ends directly anterior to the tail region), and the ventral region is composed of the entire gut area (it is directly inferior to the dorsal region, begins posterior to the face region and ends anterior to the tail region, before hindgut and anus). Since chromogenic ISH staining can contain some background enzyme activity, we standardized the cell counting and were able to differentiate one cell vs. a group of cells (red asterisk vs. white-dashed circle, respectively, in Supplementary Fig. S2d) using a custom ImageJ macro applied to images of embryos processed in the same ISH session. First, on clearly identifiable independent cells, we measured the smallest and biggest size (in pixels^2^) for one single positive cell. Then, we applied a threshold and adjusted the saturation to increase the contrast and segment cells from potential background. Finally, we indicated the minimal and maximal size for one independent cell on ‘Analyze Particles’ tool. The generated results were overlaid on the original image to remove possible artifacts. Number of positive cells per each region was normalized to the area in pixels^2^. The total number of cells per embryo was obtained as a sum of the four independent regions. Per each marker, *spiba* and *mmp7*, inter- and intragroup comparisons were analyzed. Intergroup comparisons were performed among the different experimental groups (Ctrl, BR^–^, SC^–^, Tail^–^) for each condition (NI vs. UTI) and per body region (face, tail, dorsal, ventral and total; Fig. 3c–l, Supplementary Fig. S2e–n). Intragroup comparisons were done between NI vs. UTI embryos, for each body region, within the same experimental group (Fig. 3e, o–r; Supplementary Fig. S2o–r). Particular care was taken to ensure that embryos from all the different groups per each condition were processed in the same batch at the same ISH session.

### Immunofluorescence and cell counting

Cleaved Caspase-3 (Asp175; CC3), XL-2 (anti-*Xenopus* leukocytes,^8^ and acetylated alpha-tubulin (Tub) antibodies were used to detect apoptotic cells,^43^ leukocytes,^8,43,83^ and peripheral nerves,^36,84^ respectively, on wild-type and *xlurp::GFP* embryos. Ctrl, BR^–^, SC^–^, Tail^–^, NC^–^ and Simv-treated embryos were fixed 1 h in MEMFA^76^ and washed three times in PBS. They were then permeabilized in PBS-T for 30 min at room temperature (RT), followed by a 1 h blocking at room temperature in PBST supplemented with 10% heat-inactivated goat serum. The embryos were then incubated overnight at 4 °C with the primary antibody (monoclonal mouse anti-XL2 at 1:1000, kindly provided by Makoto Asashima’s Lab at Tokyo University; polyclonal rabbit anti-CC3 at 1:300, Cell Signalling 9661; monoclonal mouse anti-Tub at 1:500, Sigma T7451). On the next day, they were washed six times in PBS (1 h each time, RT), before being incubated overnight with the secondary antibody (goat anti-mouse IgG conjugated with Alexa-Fluor 555; Invitrogen) at 4 °C. The following day, animals were photographed using an Olympus BX-UCB microscope under ×4 and ×10 magnification, controlled by Metamorph software. Particular care was taken to ensure that embryos from all the different groups were processed in the same batch at the same immunofluorescence session.

The number of XL2- or CC3-positive cells was quantified and normalized to the tail area in pixels^2^ using ImageJ software. Leukocytes and apoptotic cells were counted along the 2/3 posterior of the tail (Fig. 4a). To quantify number of GFP-positive cells (myeloid cells) and density of peripheral neural network in *xlurp::GFP* embryos, tails were subdivided in two independent regions: center and periphery (Fig. 4k, l). The somatic muscle or chevron-shaped myotomes composed ‘center’ region; ‘peripheral’ region was referred to the fin, both dorsal and ventral to the myotomes. Peripheral neural network was evaluated on images of stage 46–48 *xlurp::GFP* embryos that were immunoreacted against Tub, using gray-level measures (OD) as previously described.^36,84–88^ Two OD-mean values per animal were calculated after multiple measurements taken along the anteroposterior axis: one for center or somite region and one for peripheral fin. Analysis was done on raw black-and-white 8-bit images. Each measurement consisted of the mean value of the pixels of a fixed-size window. The size of the window remained constant across subjects. OD values ranging from 0 (black, no expression) to 255 (white, maximal expression). All individuals among whom comparisons are being made were produced in the same batch, treated identically for processing and imaging conditions were not changed. In addition, any potential aberrations originating from the optical system were corrected by background image subtraction.

### Next-generation sequencing

Following infection and surgeries at st. 25 (brain removal, spinal cut, or tail amputation), embryos were grown for 3 h at 21 °C, then their anterior parts, including brain (to ensure that differences in RNA-Seq signature among samples were not coming from the brain tissue itself), were amputated and flash-frozen in groups of 20. RNA-Seq analyses were performed by The MIT BioMicro Center (Boston, MA). Samples were quality controlled on a Fragment Analyzer (Advanced Analytical) to determine DV200 scores. The mRNA from 1 μg of total RNA was isolated using Illumina human/mouse/rat RiboZero Gold and prepared into Illumina libraries using the Kapa RNA HyperPrep kit and 10 cycles of amplification. Final libraries were pooled, and quality controlled using qPCR (Roche LC480II) as well as the Fragment Analyzer and sequenced on HiSeq2000.

Alignments were conducted by Genotypic Technology (India). Eight samples were aligned to the reference transcriptome Xenopus_laevis_v2, downloaded from NCBI [https://www.ncbi.nlm.nih.gov/genome/?term=xenopus+laevis]. There were two biological replicates per treatment (brain intact, not infected or Ctrl NI; brain intact infected or Ctrl UTI; brainless, not infected or BR^–^ NI; brainless, infected or BR^–^ UTI). The raw data from Illumina were checked for quality using FastQC1 and pre-processed, which included removing the adapter sequences and removing the low-quality bases (<q30). Pre-processing of data was done with Cutadapt2. Mapping was performed with HISAT2 to align the high-quality data to the reference genome with the default parameters. Reads are classified into aligned reads (which align to the reference genome) and unaligned reads. HISAT2 is a fast and sensitive alignment program for mapping next-generation sequencing reads (both DNA and RNA) to a single reference genome). Based on an extension of BWT for graphs,^89^ a graph FM index (GFM) was implemented. HISAT2 uses a large set of small GFM indexes that collectively cover the whole genome. These small indexes (called local indexes), combined with several alignment strategies, enable rapid and accurate alignment of sequencing reads. This new indexing scheme is called a Hierarchical Graph FM index (HGFM).

Cufflinks was used to calculate transcript abundance. It results in normalized read count in the form of FPKM values. FPKM is a unit of measuring gene/transcript expression. Four comparisons were conducted (1) Ctrl NI vs. Ctrl UTI, (2) BR^–^ NI vs. BR^–^ UTI, (3) Ctrl NI vs. BR^–^ NI, and (4) Ctrl UTI vs. BR^–^ UTI. Cufflinks includes a script called “Cuffmerge” that can be used to merge several Cufflinks assemblies together. The main purpose of this script is to develop an assembly GTF file suitable for use with Cuffdiff. Merged GTF files produced by Cuffmerge were used as input in Cuffdiff. Cuffdiff was used to calculate the differentially expressed transcripts and generated *p*-values, FDR corrected *p*-values (*q* value) and the log_2_fold change values. RNA-Seq unprocessed and processed data have been deposited in the National Center for Biotechnology Information (NCBI) Gene Expression Omnibus (GEO) and is accessible through GEO Series accession number GSE119729. Supplementary Data 1 contains all processed transcript data, including fold change, *p*-value, and *q*-value.

Sub-network enrichment analysis (SNEA) was used to identify networks enriched in each of the four comparisons. Analyses were conducted in Pathway Studio 10.0 (Elsevier) using the ResNet 11.0. The number of gene symbols that mapped to the mammalian homologs in the program ranged from ~12,300 to 12,500 for each of the four datasets using the official gene Name + Alias. Duplicates were addressed using the default “best *p*-value if present, or maximum fold change” in Pathway Studio. Sub-networks related to cell process were queried and there were 1000 permutations of the data using Kolmogorov–Smirnov algorithm to generate the distributions. SNEA identifies gene networks related to cellular processes or diseases that change with a treatment or disease; these networks are pre-defined molecular networks (expression patterns, binding, or involvement in common pathways) based upon the literature that are focused around gene hubs. All transcripts identified by RNA-seq were used in the analysis and acted as the background list for enrichment. See Supplementary Methods 1 for Reads and quality of sequencing.

### Dopamine functional assays

Liquid chromatography–mass spectrometry (LC–MS/MS): UTI-infected Ctrl and BR^–^ embryos were harvested at early st. 35 (~20 h post-surgery, 44 h post infection) immediately before the peak of infection-induced death in BR^–^ animals and the significant differences both in survival rate and apoptosis are reached respect to Ctrl embryos (experimentally defined in advance; see Fig. 2a, b). Three biological replicates per experimental group were analyzed for DA level quantification. Each replicate consisted of 30 embryos. Their anterior parts, including brain, were amputated to ensure that differences in DA levels among samples were not coming from the brain tissue itself, and then flash-frozen in groups of 30 embryos. LC–MS/MS was performed by The Small Molecule Mass Spectrometry in the Faculty of Arts and Sciences at Harvard University (Cambridge, MA). LC–MS grade dopamine hydrochloride (DA) and Dopamine-1,1,2,2-d4 hydrochloride (d4) were purchased from Millipore Sigma (cat. D-081, 73483). The working standard (DA, 0.61 ng/mL) and working internal standard solutions (DA-d4, 50 ng/mL) were diluted in LC-MS grade water. Sample preparation and protein precipitation were performed by adding 50 μL of 100 pg/μL of DA-d4 and 200 μL of cold methanol. The LC–MS/MS analysis was done on an Agilent 6460 Triple-quad mass spectrometer (Agilent Technologies) coupled to an Agilent 1290 uHPLC. The chromatographic separation was performed using an Agilent Eclipse XDB (2.1 mm internal diameter × 50 mm length × 2.7 μm particle size) C18 column. A constant flow rate of 0.400 L/min was used, along with a 10 μL injection volume. Mobile phase A was 0.1% formic acid in water v/v and B was 0.1% formic acid in acetonitrile v/v. Initial conditions were 98% A, 2% B. Compounds were eluted by increasing mobile phase composition to 98% B over 5 min, and the columns re-equilibrated to starting conditions for 3 min prior to the next injection. Targeted analysis using multiple reaction monitoring (MRM) monitoring two transitions for DA and DA-d4 using the following transitions: DA: 154→137 and 154→91, and DA-d4: *m*/*z* 158→141 and 158→95. The optimized collision energy was 9 and 25 V, respectively, for the first and second transition. A fragmentor value of 90 V was employed. The standard curve was measured between 0.1 pg/μL and 50 pg in water. The quantitative analysis was performed in MassHunter Quantitative Analysis software (Agilent Technologies, Santa Clara, USA). Data were collected from compounds with a S/N ratio of >10. A linear curve fit with a 1/x weighting was used. See Supplementary Methods 2 for LC–MS/MS conditions and parameters.

Drug Exposure. Uninfected and UTI-infected Ctrl and BR^−^ *Xenopus* embryos were exposed to specific pharmacological agents, targeting the type-1 or type-2 family of dopamine (DA) receptors (D1R, D2R), from st. 25 (immediately after brain removal) to st. 48. The drugs were refreshed every day. We used specific agonists and antagonists of each dopamine receptor: 10 μM SKF-38393, D1R agonist (SKF, Tocris 0922); 10 μM SCH-23390, D1R antagonist (SCH, Tocris 0925); 10 μM Quinpirole, D2R agonist (Quin, Tocris 1061); 1 μM L-741,626, D2R antagonist (L741, Tocris 1003). All drug treatments were performed using embryos from mixed batches of fertilizations and using three biological replicates per drug. Each replicate consisted of 30 embryos. Stock solutions of SKF, SCH and Quin were created by dissolving the compound in Millipore water to a final drug concentration of 100 mM for both SCH and Quin, and to 25 mM for SKF. Stock solution of L741 was created by dissolving the compound in DMSO to a final drug concentration of 100 mM. All aliquots were stored at −20 °C. Further dilution of all compounds was made in 20 ml of normal frog media (0.1X MMR). Control experiments were performed using embryos in 0.1X MMR (no drug), both for Ctrl and BR^−^ groups. Drug concentrations were determined through toxicity screens and were applied at levels that did not result in lethality or observable developmental defects.

### Statistics

All statistical analysis was performed using GraphPad Prism (GraphPad Software, Inc., CA, USA). Animal numbers were chosen based on previous studies as well as sample size estimates given a type I error rate of 5% and power of 0.8. Each dish of tadpoles was considered a replicate. After applying Bartlett’s test (for equal variances), data from various replicates and multiple independent groups were analyzed by one-way or two-way ANOVA test (if equal variances) or Kruskal–Wallis test (if significantly different variances). When *P* < 0.05, post hoc multiple comparisons were performed by Bonferroni or Dunn test, respectively. The significance level (α) was set to 0.05 in all cases. *P* value on graphs is after post hoc Bonferroni or Dunn analysis. The statistical values are reported as mean ± SD. Number of replicates (*r*), number of animals per replicate (*n*), number of total animals (*N* = *r* × *n*), and specific analysis used for each experiment are stated in the “Results” section and/or figure legends.

### Reporting summary

Further information on research design is available in the Nature Research Reporting Summary linked to this article.

## Supplementary information

Supplementary Information

Supplementary Data 1

Supplementary Data 2

reporting summary

Supplementary Video

## Data Availability

The authors declare that all data supporting the findings of this study are available within the article and its Supplementary Information Files or from the corresponding author upon reasonable request. RNA-seq data have been deposited into the NCBI Gene Expression Omnibus (GEO) database (Accession number: GSE119729).
