# Supplementary material for: An in vivo brain–bacteria interface: the developing brain as a key regulator of innate immunity
Source: NPJ Regen Med. 2020 Feb 4;5:2. doi: 10.1038/s41536-020-0087-2 (PMC7000827; doi:10.1038/s41536-020-0087-2)
Supplement: Supplementary file 1 — Supplementary Information [file 41536_2020_87_MOESM1_ESM.pdf]

Supplementary Figures

Supplementary Figure S1. Brain Removal, Spinal-Cord resection, Simvastatin-treated and Neural-Crest ablation

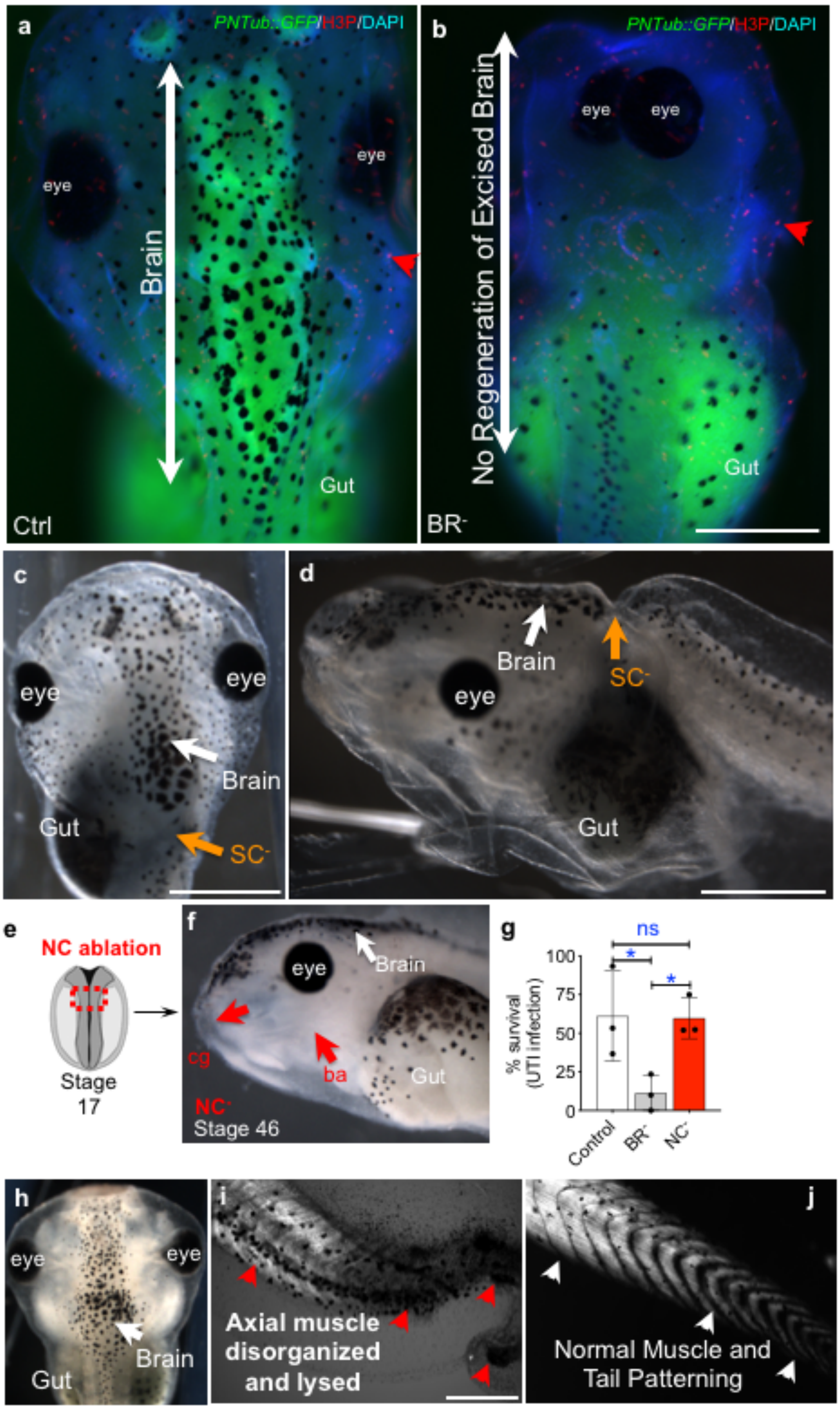

a,b. Photomicrographs of Control (Ctrl, a) and Brainless (BR⁻, b) stage-48 embryos from the transgenic line *PNTub::GFP*. To reveal potential differences in proliferative cells at the face region, we performed immunofluorescence on these embryos against the

Histone H3 phosphorylated at serine 10 (H3P, red dots pointed by red arrow), a mitosis marker extensively used in Xenopus. Our results in PNTub::GFP brainless embryos show no growth of GFP-labeled tissue at the brain area, indicating absence of brain regeneration after the complete removal (compare white arrows in a indicating the brain and its extension vs. b with no regenerated tissue along the place where the brain should be). These embryos do not display changes in H3P-positive cells at this face area (157±39 for Ctrl and 168±32 for BR^{-}; unpaired *t*-test *P*=0.76), suggesting there is no difference in the proliferative response targeted to regenerate the brain. ***c, d***. Dorsal (c) and lateral (d) views of a late-staged embryo after resection of a constant portion of spinal cord (SC), cervical levels, at stage 25. Orange arrows point the place for surgery removal, showing complete discontinuity between brain and spinal cord. ***e-g***. Neural Crest (NC) ablation assays performed on stage-17 embryos (e, red-dashed square indicates the removed NC domain) and the subsequent aberrant phenotype for the craniofacial elements (f), as defects in branchial arcs (ba) or cement gland (cg). *g*. Survival rates of Ctrl, BR^{-} and NC^{-} infected embryos. Data represent the mean and S.D. of three independent replicates (n=40, N=120). Each replicate is shown by one dot. One-way ANOVA P<0.05. Significant *P* values after Bonferroni post-hoc test are indicated as *P<0.05, ns P> 0.05. ***h-j.*** Dorsal (h) and lateral (i) views of a late-staged embryo after treatment with 0.4 uM Simvastatin for 18 hours, showing severe muscle and tail phenotype (red arrows) but normal brain morphology (white arrow). Photomicrographs after polarized light of the posterior region of the tail in an intact or control (j). a-c, h: rostral is up. d, f, i ,j: rostral is left, dorsal is up. Eyes, gut, and branchial arches (ba) are indicated for reference.

Supplementary Figure S2. The number and distribution of primitive myeloid precursors (*spiba*⁺) is not altered by presence/absence of a brain during development, with or without infection.

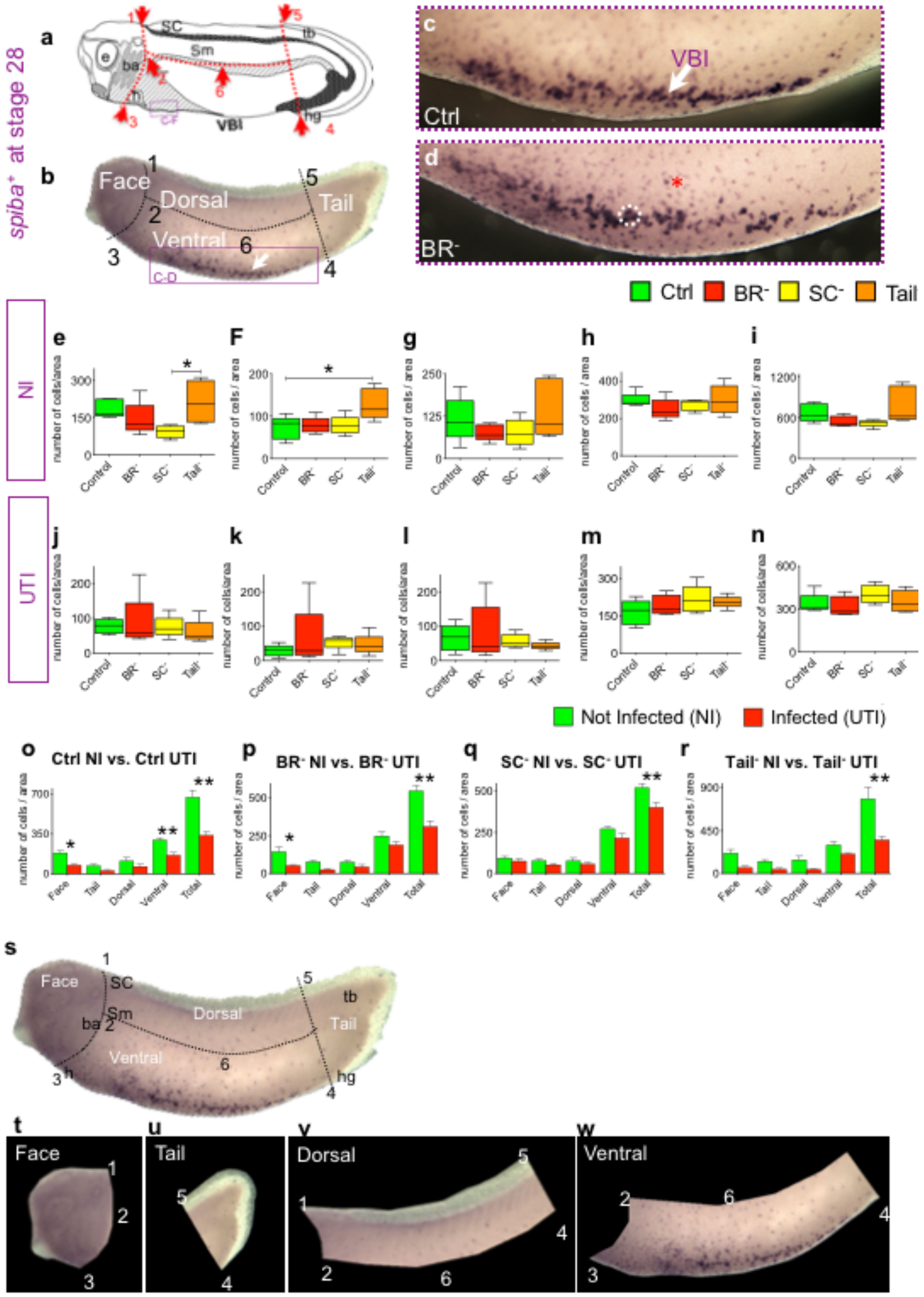

a, b. Drawing of (a) and image after *in situ* hybridization (ISH) for *spiba*⁺ (b) of one st. 28 Xenopus embryo indicating the landmarks (1-6) for the body subdivision in the four

independent areas for quantification. Dashed-purple line square in b indicates the area shown in c&d images. e:eye, ba: branchial archs, SC: spinal cord, Sm: Somite region, tb:tail bud, hg: hind gut and anus. VBI: Ventral Blood Islands or primitive hematopoietic organ. ***c, d***. Lateral view of ventral region of representative images for Control (Ctrl, c) and Brainless (BR^-, d) embryos, after *spiba* ISH. White arrow in c points VBI (same place than white arrow in b). In d, red asterisk indicates one single cells vs. white dashed-line rounding cells clumped together. Rostral is left, dorsal is up. ***e-n***. Quantification of number of *spiba*+ cells in not-infected (NI, e-i) and infected (UTI, j-n), belonging each experimental group: Ctrl (green), BR^- (red), spinal cord resection (SC^-; yellow) and Tailless ($Tail^-$; orange). Values for normalized number of *mmp7*$^+$ cells are plotted per each region and group: face (e,j), tail (f,k), dorsal (g,l) and ventral (h, m). Total (i,n) is the sum of the four regions. One-way ANOVA *P* value showed significance for e and f ($P<0.05$). ***o-r***. Number of *spiba*-positive cells (normalized to the area) in NI (green) vs. UTI (red) embryos belonging Ctrl (o), BR^- (p), SC^- (q), and $Tail^-$ (r) groups. Data represent the mean and S.D. of three independent replicates Two-way ANOVA showed significant $P<0.05$ for all comparisons. Significant *P* values after post-hoc Bonferroni comparisons are indicated as *$P<0.05$, **$P<0.01$. ***s-w***. Areas for *spiba* and *mmp7* quantification. Stage-28 embryo after ISH against *spiba* probe (s). Cell counts were performed on four independent and not-overlapping regions: face (t), tail (u), dorsal (v) and ventral (w) defined by six landmarks 1. Beginning of SC, cervical level; 2. Intersection between posterior edge of the ba and ventral edge for the first and most anterior Sm; 3. Anterior to the h; 4. End of hg; 5. Beginning of the tb; 6. ventral line formed by somites.

Supplementary Figure S3. Number and distribution of myeloid precursors (*xlurp*⁺) respect to peripheral nerve network (Tub⁺ fibers) in Control (Ctrl), Brainless (BR⁻), Spinal-Cord resection (SC⁻) and Tailless (Tail⁻) embryos.

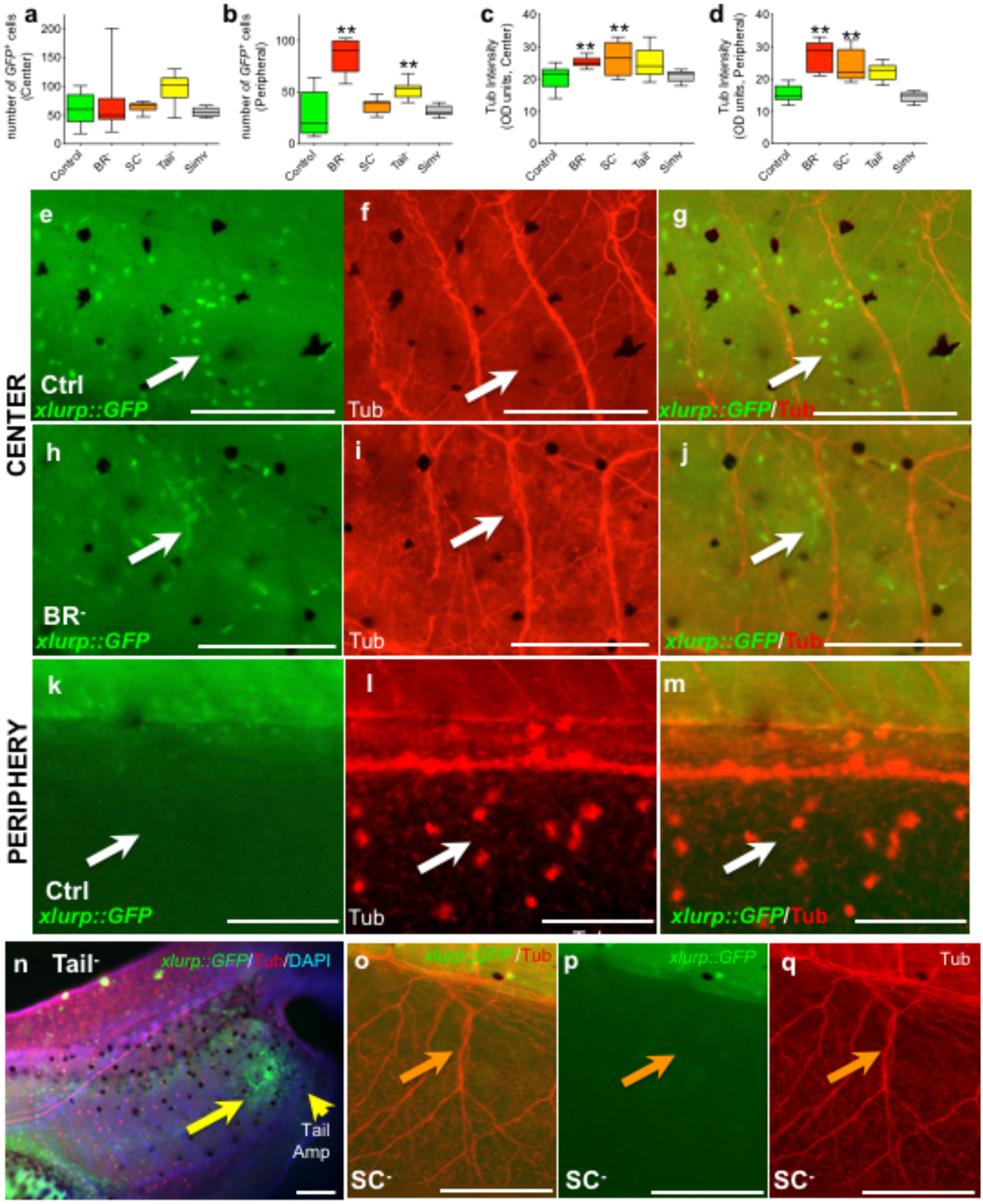

a–d. Number of GFP+ cells (normalized to the area; a,b) and peripheral nerve intensity (measured as Tub OD; c, d) for st. 48 xlurp::GFP Control, BR⁻, SC⁻, Tail⁻ and

Simv groups. *P*<0.01 for Kruskal-Wallis test. *P* values after post-hoc Dunn test, when compared to Ctrl group, is indicated as ** *P*<0.01. Data represent the mean and S.D. of, at least, ten embryos from three independent replicates. ***e–m.*** High-magnification images of center (e-j) and peripheral (k–m) tail areas in Control (e–g, k–m) and BR^{-} (h–j) *xlurp::GFP* embryos. Left column shows GFP+ myeloid cells, middle column shows neural network in red and right column shows co-location of immune cells and nerves. White arrows indicate positive elements at the same point on the three images of a row. **n–q**. Low-magnification lateral views of xlurp::GFP st. 48 $Tail^{-}$ (n) and SC^{-} (o-q) embryos after whole-mount immunofluorescence with acetylated alpha-tubulin (Tub, red), showing the myeloid cells (GFP+, green), the neural network (Tub+, red), and the co-location for immune cells and nerves (nuclei are blue after DAPI staining in N). Yellow arrows in n indicate accumulation of immune cells at the bud or amputation plane. Orange arrows in o-q indicate the same point on the three images, noting an aberrant sprouted neural network (red) but without parallel ectopic pattern for macrophages (green; as detected in BR^{-} animals, compare to Fig. 4m-o). All: Rostral is left, dorsal is up; scale bar = 250 μm.

Supplementary Figure S4. Gene networks and cell processes unique to infection in presence or absence of a brain during development.

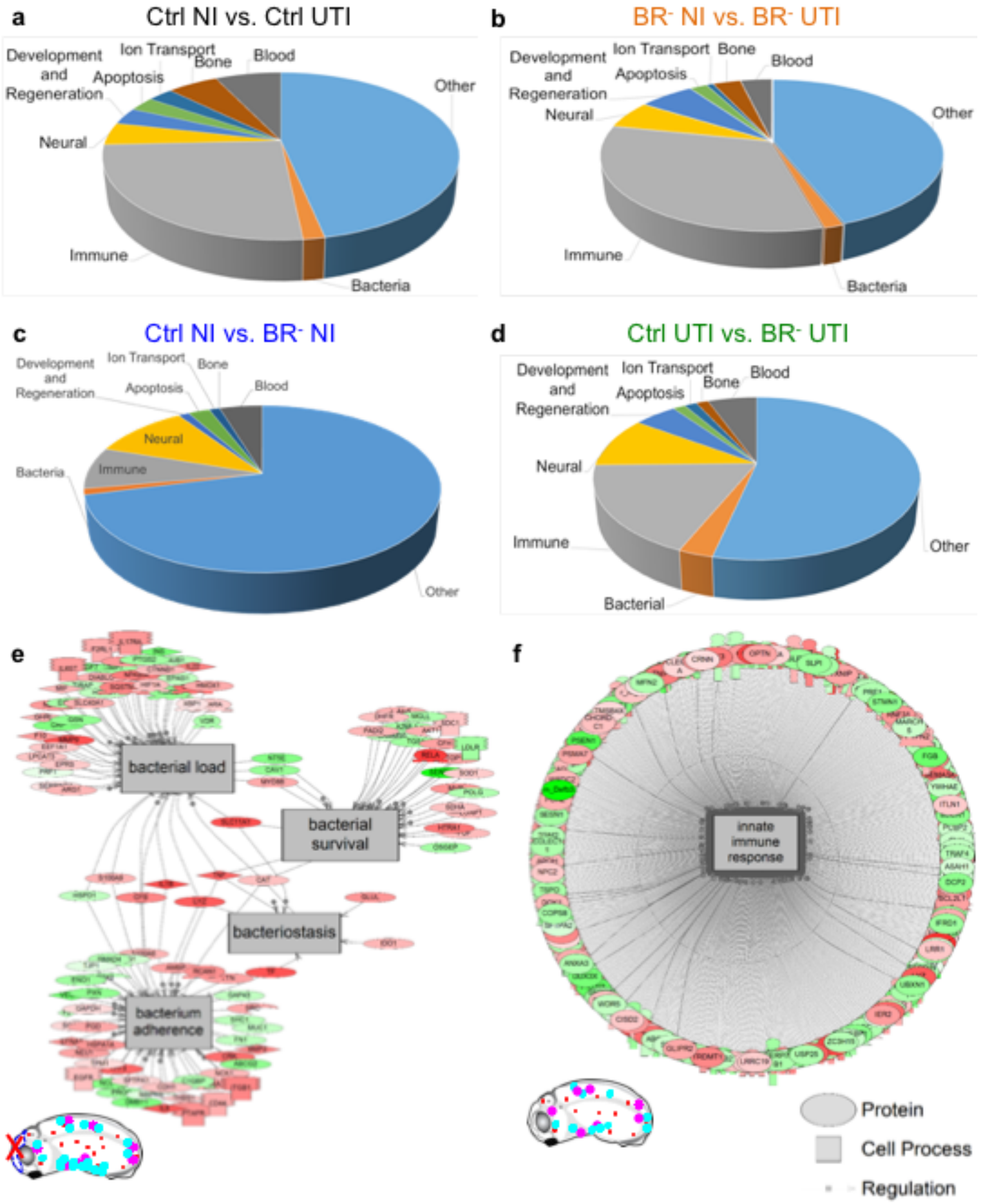

Ctrl: intact (with brain)
BR-: brainless (without brain)
NI: un-infected
UTI: infected with *E.coli*

a–d. Pie chart of the functional classification of the pathways exclusively regulated after infection in Control animals (a), after infection in BR^{-} animals (b), after brain removal without infection (c), and between infected Ctrl and infected BR^{-} embryos (d). **e.** Up-regulation of bacteria-related genes induced by infection in brainless animals. These networks were up-regulated by 20%. **f.** Innate immune response after infection in intact (or developed with a brain) animals. This pathway responds 11% less in presence of a brain than it does in absence of a brain (see Fig. 6c). Green = down gene, Red = up gene. Complete data are presented in Supplementary Data 1. All measured genes found in a pathway are located in Supplementary Data 2.

Supplementary Figure S5. Gene networks and cell processes unique to infection in presence of brain

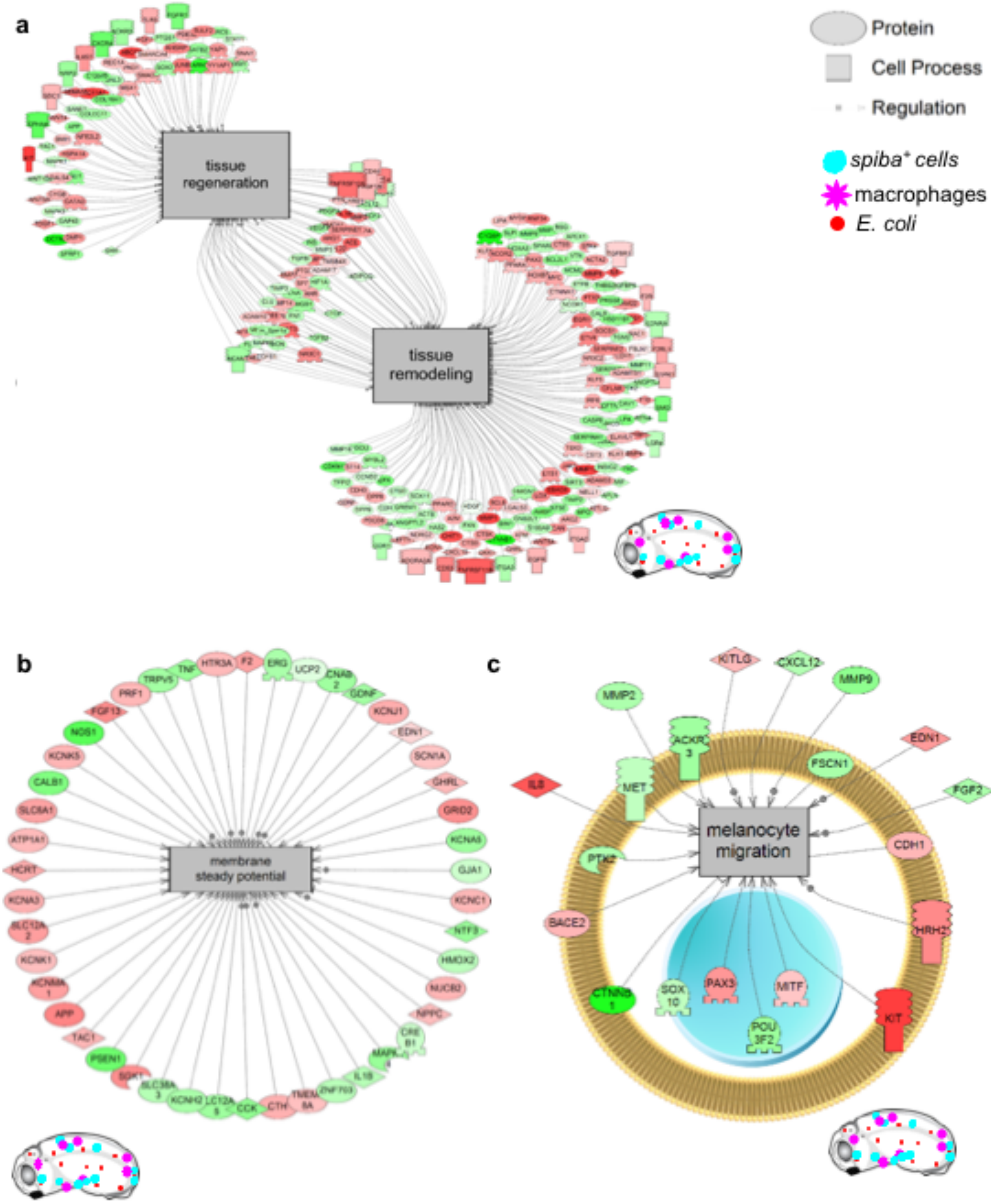

a. Considering the role of the brain on macrophages, which in turn are required for regeneration[1], we analyzed transcriptional sub-networks related to regeneration. Gene networks related to tissue regeneration/remodeling were exclusively regulated in intact animals (developing with brain) with presence of bacteria, suggesting a relationship

between brain, bacteria, and regeneration. **b.** Membrane-Potential related genes. Likewise, genes related to membrane potential are exclusively up regulated by 12%, in the presence of brain, confirming our previous results on connection between electrical signaling from the brain and slow electrical flows in the long-distance somatic tissue[2,3]. **c.** Melanocyte-migration related genes are suppressed (12%) by infection in presence of a brain. Conversely, in absence of brain, this pathway is not affected. Green = down gene, Red = up gene. Complete data are presented in Supplementary Data 1. All measured genes found in a pathway are located in Supplementary Data 2.

Supplementary Figure S6. Quantitative LC/MS/MS Analysis of Dopamine (DA) in Control (Ctrl) and Brainless (BR⁻) Xenopus embryos

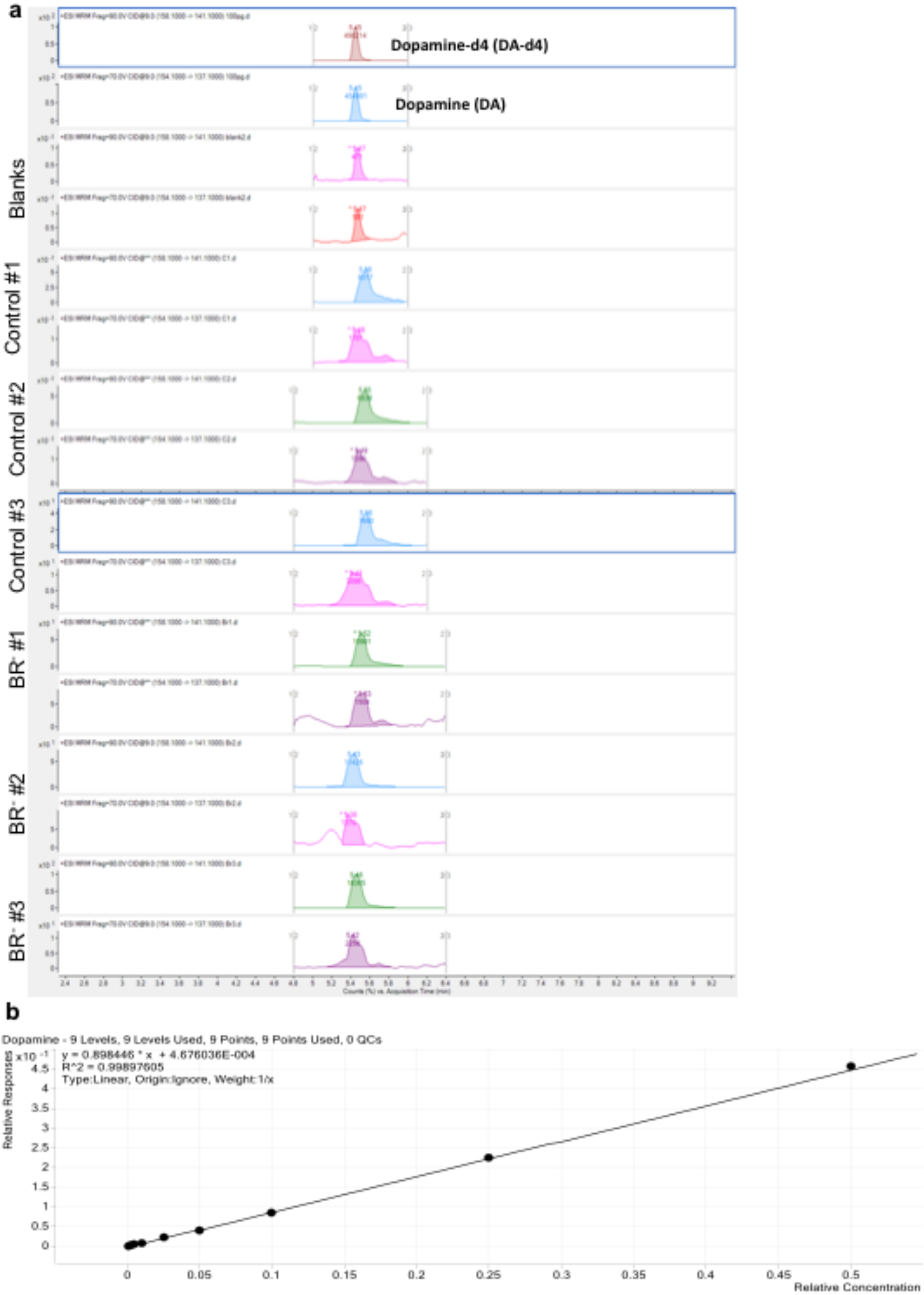

a. Chromatogram of DA, its metabolite DA-d4 and labelled versions from whole-mount embryos homogenate. **b.** Standard curve used for DA quantification, r^2=0.99897605. X-axis is concentration (μM) and Y-axis if for relative response of DA corrected by its internal standard (DA-d4).

Supplementary Tables

Supplementary Table S1. Longitudinal assay of survival rates after infection, from stage 25 (surgery) to stage 48. Data indicate the % of survival (as mean ± SD) per each stage and group

	Control (Ctrl)	Brainless (BR^{-})	Spinal Cord Resection (SC^{-})	Tail amputation ($Tail^{-}$)
st.25	100.00 ± 0.00	100.00 ± 0.00	100.00 ± 0.00	100.00 ± 0.00
st.26-27	100.00 ± 0.00	100.00 ± 0.00	100.00 ± 0.00	100.00 ± 0.00
st.30	97.07 ± 5.08	86.67 ± 5.77	88.83 ± 1.26	100.00 ± 0.00
st.35	83.70 ± 13.43	54.43 ± 12.20	71.07 ± 7.46	90.70 ± 4.69
st.40	67.60 ± 23.20	19.33 ± 7.02	62.33 ± 17.80	86.93 ± 5.25
st.42	62.70 ± 28.58	15.07 ± 9.36	61.60 ±18.11	86.93 ± 5.25
st.48	61.60 ± 28.91	11.17 ± 11.79	59.87 ± 19.50	86.07 ± 6.14

Supplementary Table S2. The top differentially expressed genes that change in response to infection in the absence of a brain. Genes depicted are those that show greater than log2 = 2 fold change following a FDR correction (Q-value). The expression patterns of all transcripts detected with RNA-seq can be found in Supplementary Data 1

Cuffmerge Gene ID	Gene Symbol	Gene Name	log2(fold_change)	P_value	Q_value
XLOC_010272	LOC108710811	uncharacterized	-5.23	0.00005	0.010
XLOC_018210	epha4	epha4	-4.61	0.0004	0.049
XLOC_035252	LOC108704891	actin, cytoplasmic 1-like	-3.16	0.00005	0.010
XLOC_003727	LOC108706479	PC-esterase domain-containing protein 1A-like	-2.33	0.00005	0.010
XLOC_022822	LOC108696180	myoferlin	-2.27	0.00005	0.010
XLOC_035987	LOC108705532	uncharacterized	-2.27	0.00005	0.010
XLOC_002807	LOC108706524	hepatocyte growth factor activator-like	-2.22	0.00015	0.024
XLOC_023974	cbl	Cbl proto-oncogene	-2.10	0.00005	0.010
XLOC_023102	LOC108696498	beta,beta-carotene 9',10'-oxygenase-like	-2.08	0.00005	0.010
XLOC_014733	LOC108714320	terminal uridylyl transferase 4 L homeolog	-2.06	0.00005	0.010
XLOC_031018	LOC108701205	axin-2-like	-2.01	0.00005	0.010
XLOC_017228	tp63	tumor protein p63	2.02	0.00025	0.035
XLOC_015623	LOC108715642	syntaxin 6 S homeolog	2.04	0.00025	0.035
XLOC_032296	LOC108702419	hemoglobin subunit alpha-3-like	2.12	0.00035	0.045

XLOC_025052	LOC108697171	tumor necrosis factor receptor superfamily member 6-like	2.16	0.0003	0.040
XLOC_006012	fam64a	family with sequence similarity 64 member A	2.27	0.00005	0.010
XLOC_033932	hist1h4a	histone cluster 1 H4 family member a	2.36	0.00005	0.010
XLOC_017594	LOC108716413	potassium-transporting ATPase alpha chain 2-like	2.64	0.00005	0.010
XLOC_009716	LOC108711653	BCL2 associated athanogene 4 L homeolog	2.80	0.00005	0.010
XLOC_005467	LOC108708616	transmembrane serine protease 12 L homeolog	2.85	0.00005	0.010
XLOC_011725	ogdh	oxoglutarate dehydrogenase	3.57	0.00005	0.010
XLOC_019228	LOC108717383	xanthine dehydrogenase/oxidase-like	3.62	0.00005	0.010
XLOC_036434	LOC108705956	uncharacterized	4.09	0.00005	0.010

Supplementary Table S3. Number of raw and processed reads for samples

Sample_Name	Raw Reads	Processed Reads	of high quality reads retained
1205	16423892	16325882	99.4032
1206	19260926	19164350	99.4985
1207	15505149	15421306	99.4592
1208	12900090	12828083	99.4418
1209	15980771	15902462	99.5099
1210	16742596	16667328	99.5504
1211	16454591	16370240	99.4873
1212	17797100	17704942	99.4821

Supplementary Table S4. Alignment statistics to the reference genome

Sample_Name	% Aligned
1205	85.16
1206	85.23
1207	82.70
1208	83.86
1209	84.82
1210	87.05
1211	86.77
1212	84.31

Supplementary Table S5. Number of expressed transcripts

Sample_Name	Transcript Expressed*	Transcript with > 1 FPKM	Genes Expressed**
1205	52581	22768	29366
1206	52921	22800	29733
1207	52359	22297	29265
1208	51637	22282	28966
1209	52108	22167	29108
1210	52469	22109	29322
1211	52850	22639	29665
1212	53010	22786	29770

* Transcripts with non-zero FPKM values ** Genes with non-zero FPKM values

Supplementary Table S6. The top differentially expressed genes in an intact control embryo after infection. Genes depicted are those that show greater than log2 = 3 fold change following a FDR correction (Q-value). The expression patterns of all transcripts detected with RNAseq can be found in Supplementary Data 1.

Cuffmerge Gene ID	Gene Symbol	Gene Name	log2 (fold_change)	P_value	Q_value
XLOC_022556	LOC108695443	WASH complex subunit 5 S homeolog	-3.95	0.0001	0.008
XLOC_030217	hba2	hemoglobin subunit alpha 2 L homeolog	-3.93	0.00005	0.004
XLOC_032296	LOC108702419	hemoglobin subunit alpha-3-like	-3.59	0.00005	0.004
XLOC_031336	hbe1	hemoglobin subunit epsilon 1 L homeolog	-3.35	0.00005	0.004
XLOC_001932	prtn3	proteinase 3 L homeolog	-3.33	0.00035	0.023
XLOC_031167	LOC108701350	cordon-bleu protein-like 1	-3.06	0.00005	0.004
XLOC_033969	tnfrsf12a	TNF receptor superfamily member 12A	3.15	0.00005	0.004
XLOC_036151	LOC108705686	uncharacterized	3.17	0.00005	0.004
XLOC_028735	LOC108699853	metalloproteinase inhibitor 1-like	3.18	0.00005	0.004
XLOC_021351	LOC108719391	tumor necrosis factor receptor superfamily member 11b L homeolog	3.21	0.0004	0.025
XLOC_023763	LOC108696179	protocadherin-8-like	3.22	0.00035	0.023
XLOC_027819	MGC84886	nuclear factor of kappa light polypeptide gene enhancer in B-cells inhibitor, alpha S homeolog	3.26	0.00005	0.004
XLOC_033289	LOC108703231	NADPH oxidase organizer 1 S homeolog	3.27	0.0006	0.034
XLOC_008408	f7	coagulation factor VII	3.27	0.00005	0.004
XLOC_004011	LOC108706768	growth-regulated alpha protein-like	3.28	0.00085	0.045
XLOC_001507	tnip2	TNFAIP3 interacting protein 2	3.28	0.00005	0.004
XLOC_000907	LOC108715061	KH and NYN domain containing L homeolog	3.29	0.00005	0.004
XLOC_010979	LOC108711587	caspase-7-like	3.30	0.00005	0.004
XLOC_015074	slc26a6	solute carrier family 26, member 6	3.34	0.0001	0.008
XLOC_008154	LOC108709471	E74 like ETS transcription factor 3 S homeolog	3.49	0.00005	0.004

XLOC_016075	rag2	recombination activating gene 2	3.57	0.00005	0.004
XLOC_028037	stom	stomatin	3.57	0.0004	0.025
XLOC_022421	cebpd	CCAAT/enhancer binding protein (C/EBP), delta	3.63	0.00005	0.004
XLOC_034120	LOC108703954	interferon-induced very large GTPase 1-like	3.74	0.00005	0.004
XLOC_029952	LOC108701277	uncharacterized	3.84	0.00005	0.004
XLOC_034935	LOC108704637	uncharacterized	3.90	0.00005	0.004
XLOC_003894	LOC108706649	neuropeptide Y receptor type 2-like	3.93	0.00005	0.004
XLOC_008262	LOC108709575	zinc finger CCCH-type containing 12A S homeolog	3.93	0.00005	0.004
XLOC_021242	LOC108719274	cytochrome P450 family 7 subfamily B member 1 L homeolog	4.11	0.00005	0.004
XLOC_026924	LOC108698357	ES1 protein homolog, mitochondrial-like	4.17	0.00005	0.004
XLOC_007356	LOC108709667	NF-kappa-B inhibitor zeta-like	4.19	0.00005	0.004
XLOC_034448	LOC108704305	tumor necrosis factor receptor superfamily member 12A-like	4.26	0.00025	0.017
XLOC_009815	LOC108711697	intercellular adhesion molecule 5-like	4.36	0.00005	0.004
XLOC_017373	LOC108717116	ST6 beta-galactosamide alpha-2,6-sialyltranferase 1 L homeolog	4.39	0.00005	0.004
XLOC_032162	LOC108702967	TRAF family member associated NFKB activator	4.40	0.00005	0.004
XLOC_026023	LOC108698598	cdc42 effector protein 2-like	4.52	0.00005	0.004
XLOC_022542	tnfrsf11b	TNF receptor superfamily member 11b	4.56	0.0002	0.014
XLOC_027554	LOC108699327	receptor-interacting serine/threonine-protein kinase 3-like	4.65	0.00005	0.004
XLOC_013524	LOC108714143	mixed lineage kinase domain-like L homeolog	4.66	0.00005	0.004
XLOC_008663	mmp8	matrix metallopeptidase 8	4.79	0.00005	0.004
XLOC_001803	LOC108711085	interleukin-8-like	4.82	0.00015	0.011
XLOC_024328	LOC108696828	hepcidin antimicrobial peptide 2 L homeolog	4.84	0.00005	0.004
XLOC_019037	tf	transferrin	4.85	0.00005	0.004
XLOC_036131	LOC108705666	yrdC domain-containing protein, mitochondrial-like	4.87	0.0002	0.014

XLOC_032 660	ada	adenosine deaminase	4.90	0.00005	0.004
XLOC_001 804	cxcl8	C-X-C motif chemokine ligand 8	5.41	0.00005	0.004
XLOC_034 817	LOC108704510	uncharacterized	5.55	0.00005	0.004
XLOC_025 787	LOC100490918	probable alpha-ketoglutarate-dependent hypophosphite dioxygenase-like	5.69	0.0004	0.025
XLOC_019 410	tnfaip3	TNF alpha induced protein 3	5.80	0.00005	0.004
XLOC_030 559	LOC108700769	colony stimulating factor 3 L homeolog	6.35	0.00065	0.037
XLOC_026 914	LOC108698345	cis-aconitate decarboxylase-like	7.14	0.0002	0.014
XLOC_014 278	tlrs5	soluble toll-like receptor 5	7.31	0.00005	0.004
XLOC_027 190	olfm4	olfactomedin 4	7.49	0.00005	0.004
XLOC_006 731	mmp1	matrix metallopeptidase 1	7.55	0.00005	0.004
XLOC_026 210	LOC108698790	tumor necrosis factor alpha-induced protein 2-like	8.71	0.00005	0.004

Supplementary Table S7. The top differentially expressed genes in brainless animals following infection. Genes depicted are those that show greater than log2 = 3-fold change following a FDR correction (Q-value). The expression patterns of all transcripts detected with RNAseq can be found in Supplementary Data 1.

Cuffmerge Gene ID	Gene Symbol	Gene Name	log2 (fold_change)	P_value	Q_value
XLOC_018210	epha4	Eph receptor A4	-4.10	0.00005	0.003
XLOC_010272	LOC108710811	uncharacterized	-3.53	0.00005	0.003
XLOC_006014	LOC108708048	uncharacterized protein C6orf132-like	-3.15	0.00005	0.003
XLOC_010979	LOC108711587	caspase-7-like	3.02	0.00015	0.009
XLOC_029907	parp14	poly(ADP-ribose) polymerase family member 14	3.04	0.00005	0.003
XLOC_011758	anxa4	annexin A4	3.04	0.00005	0.003
XLOC_023666	LOC108696094	HtrA serine peptidase 1 L homeolog	3.06	0.00015	0.009
XLOC_023319	napsa	napsin A aspartic peptidase	3.09	0.00005	0.003
XLOC_003641	prlr	prolactin receptor	3.11	0.00005	0.003
XLOC_029339	stat3	signal transducer and activator of transcription 3	3.15	0.0013	0.050
XLOC_026741	slc2a6	solute carrier family 2 member 6	3.19	0.00005	0.003
XLOC_029952	LOC108701277	uncharacterized LOC108701277	3.24	0.00005	0.003
XLOC_025591	timp-1	TIMP metallopeptidase inhibitor 1	3.24	0.00005	0.003

XLOC_036131	LOC108705666	yrdC domain-containing protein, mitochondrial-like	3.25	0.00005	0.003
XLOC_028037	stom	stomatin	3.28	0.00005	0.003
XLOC_036966	irg1	Infection Response Gene	3.28	0.00005	0.003
XLOC_015074	slc26a6	solute carrier family 26, member 6	3.32	0.00005	0.003
XLOC_005365	LOC108708498	immunoresponsive 1 homolog	3.34	0.0005	0.024
XLOC_028328	cfb	complement factor B	3.34	0.00005	0.003
XLOC_034817	LOC108704510	uncharacterized	3.40	0.00005	0.003
XLOC_003894	LOC108706649	neuropeptide Y receptor type 2-like	3.41	0.00005	0.003
XLOC_008663	mmp8	matrix metallopeptidase 8	3.45	0.00005	0.003
XLOC_032660	ada	adenosine deaminase	3.46	0.00005	0.003
XLOC_022542	tnfrsf11b	TNF receptor superfamily member 11b	3.47	0.00005	0.003
XLOC_008262	LOC108709575	zinc finger CCCH-type containing 12A S homeolog	3.56	0.00005	0.003
XLOC_021242	LOC108719274	cytochrome P450 family 7 subfamily B member 1 L homeolog	3.59	0.00005	0.003
XLOC_028735	LOC108699853	metalloproteinase inhibitor 1-like	3.60	0.00005	0.003
XLOC_034230	LOC108704035	ninjurin-1-like	3.66	0.00055	0.025
XLOC_027819	MGC84886	nuclear factor of kappa light polypeptide gene enhancer in B-cells inhibitor, alpha S homeolog	3.71	0.00005	0.003
XLOC_001780	grid2	glutamate receptor, ionotropic, delta 2	3.80	0.00015	0.009
XLOC_024328	LOC108696828	hepcidin antimicrobial peptide 2 L homeolog	3.86	0.00005	0.003
XLOC_003778	LOC733361	TNFAIP3 interacting protein 2 S homeolog	3.97	0.00005	0.003
XLOC_020434	ttr	transthyretin	4.01	0.00005	0.003
XLOC_030439	rnf40	ring finger protein 40, E3 ubiquitin protein ligase L homeolog	4.01	0.00005	0.003
XLOC_017373	LOC108717116	ST6 beta-galactosamide alpha-2,6-sialyltranferase 1 L homeolog	4.03	0.00005	0.003
XLOC_034935	LOC108704637	uncharacterized	4.05	0.00005	0.003
XLOC_026023	LOC108698598	cdc42 effector protein 2-like	4.08	0.00005	0.003
XLOC_004011	LOC108706768	growth-regulated alpha protein-like	4.08	0.00075	0.032
XLOC_013524	LOC108714143	mixed lineage kinase domain-like L homeolog	4.27	0.00005	0.003
XLOC_010282	tnip1	TNFAIP3 interacting protein 1	4.28	0.00005	0.003
XLOC_032162	LOC108702967	TRAF family member associated NFKB activator S homeolog	4.36	0.00005	0.003
XLOC_009635	LOC108711579	uncharacterized	4.36	0.00015	0.009
XLOC_026916	LOC108698348	ES1 protein homolog, mitochondrial-like	4.41	0.00005	0.003
XLOC_033289	LOC108703231	NADPH oxidase organizer 1 S homeolog	4.42	0.0004	0.020
XLOC_025426	cnfn-b	cornifelin, gene 1 S homeolog	4.53	0.00095	0.039
XLOC_026914	LOC108698345	cis-aconitate decarboxylase-like	4.67	0.00005	0.003
XLOC_001804	cxcl8	C-X-C motif chemokine ligand 8	4.97	0.00005	0.003
XLOC_009815	LOC108711697	intercellular adhesion molecule 5-like	4.99	0.00005	0.003

XLOC_013785	LOC108713547	vascular cell adhesion molecule 1 L homeolog	5.26	0.00065	0.029
XLOC_019037	tf	transferrin	5.87	0.00005	0.003
XLOC_019410	tnfaip3	TNF alpha induced protein 3	5.92	0.00005	0.003
XLOC_023188	LOC108696592	tumor necrosis factor receptor superfamily member 9 L homeolog	6.03	0.00005	0.003
XLOC_004012	LOC108706769	interleukin-8-like	6.31	0.00005	0.003
XLOC_027553	ripk3	receptor-interacting serine-threonine kinase 3	6.66	0.00045	0.022
XLOC_025785	LOC108698350	probable alpha-ketoglutarate-dependent hypophosphite dioxygenase	6.85	0.00005	0.003
XLOC_027190	olfm4	olfactomedin 4	6.89	0.00005	0.003
XLOC_014278	tlrs5	soluble toll-like receptor 5	7.51	0.00005	0.003
XLOC_026210	LOC108698790	tumor necrosis factor alpha-induced protein 2-like	7.95	0.00005	0.003
XLOC_006731	mmp1	matrix metallopeptidase 1	8.39	0.0001	0.006

Supplementary Table 8. The top differentially expressed genes that change with brain removal (in absence of infection). Genes depicted are those that show greater than log2=2-fold change following a FDR correction (Q-value). The expression patterns of all transcripts detected with RNAseq can be found in Supplementary Data 1. Common genes responsive to brain removal and tail removal in Xenopus[4] has been excluded for this analysis.

Cuffmerge Gene ID	Gene Symbol	Gene Name	log2(fold_change)	P_value	Q_value
XLOC_022556	LOC108695443	WASH complex subunit 5 S homeolog	-4.10	0.00005	0.016
XLOC_030439	rnf40-b	ring finger protein 40, E3 ubiquitin protein ligase L homeolog	-3.98	0.0001	0.029
XLOC_000096	LOC108697691	nuclear receptor binding SET domain protein 2 L homeolog	-3.33	0.00005	0.016
XLOC_026931	LOC108698366	uncharacterized	-2.82	0.00005	0.016
XLOC_035252	LOC108704891	actin, cytoplasmic 1-like	-2.80	0.00005	0.016
XLOC_022843	tbc1d12	TBC1 domain family member 12 L homeolog	-2.60	0.00005	0.016
XLOC_025638	LOC108698163	euchromatic histone-lysine N-methyltransferase 1 L homeolog	-2.56	0.00005	0.016
XLOC_011225	tyk2	tyrosine kinase 2	-2.37	0.00005	0.016
XLOC_024032	LOC108696525	agrin-like	-2.23	0.00005	0.016
XLOC_011552	akr1c8p	aldo-keto reductase family 1 member C8	-2.14	0.00005	0.016
XLOC_036933	LOC108706318	uncharacterized	2.03	0.00005	0.016
XLOC_029735	LOC108701066	acyl-CoA synthetase short chain family member 2,	2.21	0.00005	0.016

		gene 1 L homeolog			
XLOC_009229	LOC108711149	leiomodin-2-like	2.25	0.000 05	0.016
XLOC_021094	LOC108719134	neural precursor cell expressed, developmentally down-regulated 9 L homeolog	2.48	0.000 05	0.040
XLOC_006014	LOC108708048	uncharacterized protein C6orf132-like	2.56	0.000 05	0.016
XLOC_027229	LOC108698676lonrf	FBJ murine osteosarcoma viral oncogene homolog L homeolog LON peptidase N-terminal domain and ring finger 1	2.66	0.000 1	0.016
XLOC_022613XLOC_027229	add3LOC108698676	adducin 3FBJ murine osteosarcoma viral oncogene homolog L homeolog	2.88	0.000 05	0.029
XLOC_017594XLOC_022613	LOC108716413add3	potassium-transporting ATPase alpha chain 2-like adducin 3	3.02	0.000 05	0.016
XLOC_026918XLOC_017594	LOC108698346LOC108716413	cis-aconitate decarboxylase-like potassium-transporting ATPase alpha chain 2-like	3.08	0.000 05	0.016
XLOC_004116XLOC_026918	LOC108704680LOC108698346	mucin-22-likecis-aconitate decarboxylase-like	3.57	0.000 1	0.016
XLOC_025787XLOC_004116	LOC100490918LOC108704680	probable alpha-ketoglutarate-dependent hypophosphite dioxygenase-like mucin-22-like	3.87	0.000 05	0.029

Supplementary Methods

Supplementary Methods 1: Reads and quality of sequencing

From a total of 131,065,115 (50x1) reads, 130,384,593 high quality adapter free reads were utilized in subsequent analyses. On average 16,298,074 reads were retained for downstream analysis (Supplementary Table S2). For every sample, an average of 99.47% of high-quality data were retained. An average of ~83.86% of processed reads aligned to the *Xenopus* reference genome (Supplementary Table S3). There was an average of 52,491 transcripts expressed across all samples. Transcripts were identified and quantified based on aligned reads. Transcript expression was generated through Cufflinks. Compiled expression profiles at the transcript level, as well as gene level, were represented in the form of an FPKM matrix and the total number of expressed transcripts in all the samples is given in Table S4. Group-wise comparison at the transcript level was performed to identify differentially regulated transcripts between any two of the four conditions. The number of differentially expressed genes (DEGs) was determined after multiple hypothesis correction. The q value (adjusted *P*-value) was set at 0.05 and DEGs were considered to be those transcripts passing this correction (all expression data can be found in Supplementary Data 1 for all comparisons).

Supplementary Methods 2: LC/MS/MS Method for Dopamine & Dopamine-d4.

<u>LC parameters:</u>

Column: Agilent Eclipse XDB-C18 (Agilent Technologies)

Solvent (A) 0.1% Formic acid

Solvent (B) 0.1% Formic acid in acetonitrile

Flow rate: 0.4mLs/min

10uL injection

Standards / samples analyzed in H2O

<u>Reverse phase gradient:</u>

0.00min @ 2% B

6.00min @ 100% B

7.00min @ 100% B

7.10min @ 2% B

10.0min @ 2% B

Mass Spec parameters (Agilent 6460 Triple-quad LCMS System) (Agilent Technologies)

MRM (multiple reaction monitoring)

Compound	Precursor ion	Product ion	Dwell	Frag(V)	CE (V)	polarity
Dopamine	154.1	137.1	200	70	9	positive
Dopamine	154.1	91.1	200	70	25	positive
Dopamine-d4:	158.1	141.1	200	90	9	positive
Dopamine-d4:	158.1	95.1	200	90	29	positive

Gas Temp: 350C
Gas flow: 12 L/min
Nebulizer: 35 psi
SheathGasHeater: 400C
SheathGasflow: 12 L/min
Capillary(V): 4000
VCharging: 500

Supplementary Notes

Supplementary Note 1: *Xenopus* Immune System: early 30's vs. late 40's.

At st. 30, the only immunity present in embryo is driven by innate-immune cells. During the first 12 d post-fertilization (until approx. st. 47, in which differentiated B and T cells emerge in the periphery), the embryo must rely on innate type of myeloid cells or nonlymphocyte leukocytes for defense (reviewed in[5]). The thymus (where T cells differentiate) arises around st. 40 and, by st. 48, the cortex–medulla architecture becomes visible with expression of mature T-cell markers (such as MHC class II, CTX etc). The spleen anlage forms around st. 47 where lymphocytes accumulate by st. 48-49, a time that corresponds to the first detectable Ab response and detection of mature competent B cells (reviewed in[6]). Although our study focusses on myeloid cells, it is clear that the Xenopus immune system at early (around stage 30) vs. later stages (from st. 47) is driven by different players and organs[7,8], and response at stage 48 will be more complex, elaborated in terms of cell differentiation and recruitment. The embryonic anterior blood island, initial source of myeloid cells, is formed at st. 20. At the early st. 30, the myeloid cells present in embryo, 'myelocytes', are still considered primitive or serving as transient innate immune effector cells[9]. At later stages, and subsequent to the establishment of blood circulation, expression of a fluorescence marker under the control of cell type-specific promoters in transgenic animals revealed complex and diverse populations of myeloid cells including granulocytic and monocytic lineages[10] (reviewed in[11]) starting at around st. 40-46.

Supplementary Note 2: Summary of the top differentially expressed genes.

Infection in an intact embryo induced the down-regulation of genes encoding neutrophil elastase (proteinase 3) and cordon-bleu protein-like 1 which is involved in an actin-filament network. Immune- and neural-related genes were highly up regulated in infected intact animals, especially transcripts for colony stimulating factor 3 L homeolog (CSF3), tumor necrosis factor (TNF) receptor-I signaling complex, RAG complex, and protocadherin-8-like gene (Supplementary Table S6). The transcriptional outcome for brainless animals with infection was characterized for the up-regulation of genes related to bacteria metabolism (such as the probable alpha-ketoglutarate-dependent hypophosphite dioxygenase) and immune activity, such as

TNFR signaling, interleukin-8 and the immunoglobulin VCAM-1 (vascular cell adhesion molecule 1L homolog) (Supplementary Table S7). Brain removal (without infection, Table S8, and after subtracting transcripts in common with tail removal) specifically induced high up-regulation of genes related to genome instability and neural development. Some of the common DEGS for unspecific removal of any organ of the body were *fos* or *lonrf1*. Highest DEGs displayed in the response to infection in absence of brain (Ctrl UTI vs. BR^{-} UTI; Supplementary Table S2) included down-regulation of transcripts related to tissue injury and repair (such as myoferlin and growth factors), ubiquitination of proteins, RNA urydylation and stability of beta catenin. The most up-regulated genes were related to BAG-4 proteins, cytokine receptors and TNFR pathway.

Supplementary Data

Supplementary Data 1. List of all genes and subnetworks differentially expressed between two conditions.

Supplementary Data 2. List of genes and acronyms used in the subnetwork enrichment analysis of the transcriptome for not-infected (NI) and infected (UTI) control and brainless embryos.

Supplementary Video S1. Time-lapse movie of a *Xlurp::GFP* embryo at stage 26, showing real-time macrophage behavior. Duration 3h 30 min, 5 fps.

References for Supplementary Information

1 Godwin, J. W., Pinto, A. R. & Rosenthal, N. A. Macrophages are required for adult salamander limb regeneration. *Proceedings of the National Academy of Sciences* **110**, 9415-9420, doi:10.1073/pnas.1300290110 (2013).

2 Herrera-Rincon, C., Pai, V. P., Moran, K. M., Lemire, J. M. & Levin, M. The brain is required for normal muscle and nerve patterning during early Xenopus development. *Nature communications* **8**, doi:10.1038/s41467-017-00597-2 (2017).

3 Pai, V. P., Lemire, J. M., Chen, Y., Lin, G. & Levin, M. Local and long-range endogenous resting potential gradients antagonistically regulate apoptosis and proliferation in the embryonic CNS. *The International journal of developmental biology* **59**, 327-340, doi:10.1387/ijdb.150197ml (2015).

4 Chang, J., Baker, J. & Wills, A. Transcriptional dynamics of tail regeneration in Xenopus tropicalis. *Genesis (New York, N.Y. : 2000)* **55**, doi:10.1002/dvg.23015 (2017).

5 Hansen, J. D. & Zapata, A. G. Lymphocyte development in fish and amphibians. *Immunological reviews* **166**, 199-220 (1998).

6 Robert, J. & Ohta, Y. Comparative and developmental study of the immune system in Xenopus. *Dev Dyn* **238**, 1249-1270, doi:10.1002/dvdy.21891 (2009).

7 Imai, Y. *et al.* Multiple origins of embryonic and tadpole myeloid cells in Xenopus laevis. *Cell Tissue Res* **369**, 341-352, doi:10.1007/s00441-017-2601-4 (2017).

8 Ciau-Uitz, A., Liu, F. & Patient, R. Genetic control of hematopoietic development in Xenopus and zebrafish. *Int J Dev Biol* **54**, 1139-1149, doi:10.1387/ijdb.093055ac (2010).

9 Costa, R. M., Soto, X., Chen, Y., Zorn, A. M. & Amaya, E. spib is required for primitive myeloid development in Xenopus. *Blood* **112**, 2287-2296, doi:10.1182/blood-2008-04-150268 (2008).

10 Paredes, R., Ishibashi, S., Borrill, R., Robert, J. & Amaya, E. Xenopus: An in vivo model for imaging the inflammatory response following injury and

bacterial infection. *Dev Biol* **408**, 213-228, doi:10.1016/j.ydbio.2015.03.008 (2015).

11 Grayfer, L. & Robert, J. Amphibian macrophage development and antiviral defenses. *Developmental and comparative immunology* **58**, 60-67, doi:10.1016/j.dci.2015.12.008 (2016).
